# Adult Chinese Spanish L2ers’ acquisition of phi-agreement and temporal concord: The role of morphosyntactic features and adverb/subject-verb distance

**DOI:** 10.3389/fpsyg.2022.1007828

**Published:** 2022-12-22

**Authors:** Tiaoyuan Mao, Nicoletta Biondo, Zilong Zheng

**Affiliations:** ^1^Institute of Linguistics, Beijing Foreign Studies University, Beijing, China; ^2^Department of Psychology, University of California, Berkeley, Berkeley, CA, United States; ^3^Basque Center on Cognition, Brain, and Language, San Sebastian, Spain; ^4^School of Foreign Languages, Soochow University, Suzhou, China

**Keywords:** morphosyntax, Chinese Spanish L2ers, subject-verb agreement, adverb-verb temporal concord, processing mechanisms, self-paced reading

## Abstract

**Introduction:**

While phi-agreement and concord are suggested to differ in nature during the first language (L1) acquisition, the acquisition of adverb-verb TC and SV person/number agreement by Chinese Spanish second language (L2) learners has only received limited attention. The current study examined morphosyntactic processing by advanced Chinese Spanish L2 learners (L2ers), whose L1 lacks the explicit morphological marking of tense and phi-agreement.

**Method:**

Chinese Spanish L2ers and native Spanish speakers were asked to complete a self-paced grammaticality judgment task, where the grammaticality of adverb-verb TC and SV person/number agreement as well as the adverb/subject-verb distance were manipulated.

**Results:**

For both native Spanish speakers and L2ers, SV agreement violations are detected earlier and judged more accurately than adverb-verb TC violations. Furthermore, L2ers process SV number agreement less efficiently than SV person agreement (but as efficiently as adverb-verb TC). And there is no influence of the adverb/subject-verb distance on the processing of verbal inflection.

**Conclusions:**

This study suggests that advanced Chinese Spanish L2ers tend to use native-like cognitive mechanisms for phi-agreement and concord computations, though their sensitivity to agreement violations may be further influenced by the morphosyntactic feature involved.

## 1. Introduction

Morphosyntactic variation is regarded as a typical landmark during the acquisition of verbal inflection in the first and second language acquisition (*cf.* Rice et al., 1998; Lardiere, 2000; Prévost and White, 2000; Mao, 2020b; Chomsky, 2023). A testing ground for smooth morphosyntactic development in language acquisition is the investigation of how acquirers accurately operate the mechanism of Agree, based on the features encoded in verbal morphology and related lexical items. Therefore, the exploration of agreement between syntactic constituents such as the subject and the verb, along with the temporal concord between an adverb and the verb, turns out to be an important issue in language acquisition (*cf.* Jegerski, 2016). It is because syntactic operations, such as SV agreement and TC concord, enable people to verify how agreement processing/parsing, “the third-factor computational operation” (Chomsky, 2005, p. 49; Mao, 2020a, 2021, in press), mirrors or facilitates the unfolding of language acquisition.

In theoretical linguistics, the subject-verb (SV) agreement is viewed as a syntactic or primary relation while the adverb-verb temporal concord (TC) is a discourse-related or non-primary one (Frazier and Clifton, 1996; Biondo et al., 2018). As research shows, the former is acquired early in childhood and fairly preserved in agrammatic aphasia, while the latter is acquired later and sharply impaired in agrammatic aphasia (*cf.* Friedmann and Grodzinsky, 1997; Clahsen and Ali, 2009; Belletti and Guasti, 2015). There is evidence showing that SV agreement and adverb-verb TC relations are differently processed during sentence comprehension (e.g., Fonteneau et al., 1998; Biondo et al., 2018). For example, De Vincenzi et al. (2006) conducted a self-paced reading task with native Italian speakers and found that SV number agreement violations triggered immediate costs at the target region (the verb) and at the following one (longer reading times in ungrammatical sentences than in grammatical sentences), while adverb-verb TC violations gave rise to processing costs only at the post-target region (i.e., the word following the verb).

Although different processing patterns between SV agreement and adverb-verb TC can be explained by a relation-based approach like the Construal model (Frazier and Clifton, 1996), feature’s interpretive properties (anchor) have also been investigated in a growing body of research (e.g., Bianchi, 2003; Sigurðsson, 2004; Mancini et al., 2013). In an event-related potential (ERP) study on L1 Spanish verbal inflection, Mancini et al. (2011) found that number violations elicited a LAN followed by a P600 component, while person violations yielded N400–P600 effects (but see Zawiszewski et al., 2016 for different ERP results). The different ERP patterns can be accounted for by a feature-anchoring approach, which proposes that each morphosyntactic feature needs to activate a specific link between its morphosyntactic expression and its semantic/discourse-related content (which functions as an interpretive anchor). The anchor for [Number] feature is represented by the grammatically semantic representation of the subject, which signals the numerosity of the argument of the subject (a single entity vs. a plurality). The anchor for [Person] is represented by the discourse representation, which expresses the status of the subject with respect to the participants in the speech act (e.g., speaker, addressee). Therefore, the person-number processing dissociation can be attributed to the activation of different interpretive properties (Mancini et al., 2011).

In an eye-movement study in L1 Spanish, Biondo et al. (2018) also found the person-number dissociation, by showing larger parsing costs for person violations compared to number violations at the target region (in total reading time) and at the post-target region (in first-pass, and total reading time) when the subject was adjacent to the verb, but only in the go-past duration of the post-target region when an adverb intervened between the subject and the verb. Moreover, they found larger processing costs in early and late reading measures for number/person violations compared to the correct agreement condition in both the local and distal configurations, whereas parsing costs arose in early measures for tense violations only in the distal configuration (not in the local one). Due to these processing differences, Biondo et al. (2018) argued that the feature-anchoring approach should widen its scope by including the processing of [Tense] features. Tense interpretation requires both the adverb and verb tense specifications to be anchored to discourse, which determines the temporal coordinates of the event (with reference to the speech time). Anchoring the adverb to discourse requires time. The distance that separates the adverb from the verb *via* a subject gives the parser enough time for the completion of the previous anchoring of the adverb before the same process is triggered at the verb position, resulting in an earlier emergence of tense violation effects in the distal configuration.

There are other models of sentence parsing that have been proposed to account for agreement processing by adult native speakers, but they differ in their predictions for the impact that different syntactic relations (primary and non-primary) and different morphosyntactic features (e.g., [Number], [Person], and [Tense]) may have on sentence comprehension. Indeed, some models predict a unique mechanism for the processing of different concord relations, independent from the types of relations and features under computation (e.g., Hagoort, 2003). Some models predict a difference during the processing of primary and non-primary relations, but they do not predict different parsing routines for different features (e.g., [Number] and [Person]) within the same type of relation (e.g., Frazier and Clifton, 1996). Moreover, all these parsing models focus on processing mechanisms specific to adult native speakers, but not on those in children and adult L2 learners. In other words, current models do not clearly explain how the computation of various syntactic relations and features encoded in verbal morphology influence sentential parsing, and ultimately, language acquisition (*cf.* Biondo et al., 2018, p. 2), which asks for further investigation. Given that the current study narrows down on Spanish L2 acquisition, the relevant research will thereby be examined below.

## 2. Acquisition of Spanish L2 SV agreement and adverb-verb TC

While there are many studies concerning the L2 acquisition of either SV agreement or adverb-verb TC in Spanish (e.g., Vanpatten et al., 2012; Sagarra and Ellis, 2013), only few studies have compared the processing of these two relations in the same experimental study. For instance, one ERP study investigated how English learners of Spanish with different levels of L2 proficiency processed SV (number) and TC (tense) violations (Biondo and Mancini, 2021). Preliminary results showed that English Spanish L2ers of the low and intermediate proficient groups preferred to employ “semantic-based strategies” (Clahsen and Felser, 2006; Steinhauer et al., 2009) to process TC during early stages of L2 acquisition, as reflected by a larger sustained negativity for TC violations than for the control condition in the groups with low and intermediate levels of proficiency. Conversely, the group with high levels of L2 proficiency was able to parse the formal grammatical rule, as evidenced by the results in which both SV and TC violations elicited a larger P600 compared with control conditions, with SV violations eliciting an earlier P600 than TC violations (Biondo and Mancini, 2021). The high proficient Spanish L2ers thus seem to represent a good test case for a comprehensive exploration of the mechanism(s) at play during the processing of these two relations.

Although Biondo and Mancini (2021) have demonstrated that SV agreement and adverb-verb TC were analyzed differently even for advanced English Spanish L2ers, it remains unclear whether this conclusion is generalized to L2ers whose L1 is morphologically impoverished, such as Chinese-Spanish L2ers (for a comparison of Chinese and Spanish morphological markings see next section).

Up to now, the idea that the L1–L2 similarities/differences in morphological markings impact the processing of L2 morphosyntactic properties has been further tested in other studies, most of which were conducted to compare the parsing routines of L2ers with various L1 backgrounds. For instance, by using eye-tracking to measure the acquisition of Spanish adverb-verb TC, Sagarra and Ellis (2013) found that Romanian (rich morphology) learners of Spanish (rich morphology) looked longer at incorrect verbs than English (relatively poor morphology) learners of Spanish (who might directly process the ungrammatical information). This implies that even if the same functional features might exist in the L1s of two types of L2ers, L2ers with morphologically richer L1s seem to rely more heavily on L2 morphological cues to assign the temporal reference. L1–L2 morphological similarities/differences could thus make the exploration of how Chinese-Spanish learners parse SV agreement and TC a relevant research question, given the optional use of number morphology on nouns and the absence of verb number morphology in Chinese (for a specific explanation of Chinese morphological markings see the next section).

One may contend that late L2ers can overcome the difficulties posed by the L1 and ultimately acquire native-like morphosyntactic knowledge of the non-native morphology. For example, Foote (2011) tested English learners of Spanish who acquired the L2 early or late in life. In particular, the author tested the learners’ sensitivity to Spanish inflections in a series of self-paced reading tasks and found that the two groups were sensitive to the violations of both SV number agreement (a construction shared by both L1 and L2) and noun-adjective gender agreement (a construction unique to the L2). Likewise, Yao and Chen (2017) showed that high-proficiency Chinese English L2ers are able to process [Tense], [Number], and [Person] features of English verbal inflection (e.g., the past tense-ed, the third person singular present tense *–s*). Along these lines, it seems that proficient L2 learners will provide a more convincing perspective to examine the relations.

Interestingly, the result from advanced Chinese English L2ers showed that TC violations were detected earlier than SV agreement violations (Yao and Chen, 2017), contrary to what was found for the advanced English Spanish L2ers (Biondo and Mancini, 2021). If so, it is unclear whether the contradictory results are caused by the morphological differences across the L1s or the L2s, or other factors. To address all these open questions, the current study selects the advanced Chinese Spanish L2ers as the target group. An investigation focusing on the advanced Chinese Spanish L2ers can provide a good indication of whether L2ers with a morphologically poor L1 recruit native-like mechanisms to process SV agreement and adverb-verb TC relations.

## 3. SV agreement and adverb-verb TC in Spanish and Chinese

Chinese speakers have been reported to struggle with the processing of SV number agreement in their L2, such as English (*cf.* Jiang, 2004; Lempert, 2016). The difficulty is related to the different realization of inflectional morphology in Chinese and Indo-European languages. In many Indo-European languages, finite verbs overtly agree with the subject in [Number] and [Person]. For example, in Spanish the agreement morphology is quite rich: Different morphological suffixes are used to express [Number] and [Person] on the verb, as shown in (1–3). The same applies to [Tense] as shown in (4–6).

1. a. Yo_[1st Person] [SG]_ hablo_[1st Person] [SG]_ (‘I speak’)^1^
  b. nosotros_[1st Person] [PL]_ (as) hablamos_[1st Person] [PL]_ (‘We speak’)
2. a. tú_[2nd Person] [SG]_ hablas_[2nd Person] [SG]_ (‘You (singular; informal) speak’)
  b. vosotros (as) _[2nd Person] [PL]_ habláis_[2nd Person] [PL]_ (‘You (plural; informal) speak’)
3. a. él/ella/Ud. _[3rd Person] [SG]_ habla_[3rd Person] [SG]_
  (‘He/She/It speaks – You (singular; formal) speak’)
  b. ellos (as) /Uds._[3rd Person] [PL]_ hablan_[3rd Person] [PL]_
  (‘They speak – You (plural; formal) speak’)
4. a. Ayer hablé_[1st Person] [SG] [PST]_/hablamos_[1st Person] [PL] [PST]_
  (‘Yesterday (I/we) spoke’)
  b. Mañana hablaré_[1st Person] [SG] [FUT]_/hablaremos_[1st Person] [PL] [FUT]_
  (‘Tomorrow (I/we) will speak’)
5. a. Ayer hablaste_[2nd Person] [SG] [PST]_/hablasteis_[2nd Person] [PL] [PST]_
  (‘Yesterday (you) spoke’)
  b. Mañana hablarás_[2nd Person] [SG] [FUT]_/hablaréis_[2nd Person] [PL] [FUT]_
  (‘Tomorrow (you) will speak’)
6. a. Ayer habló_[3rd Person] [SG] [PST]_/hablaron_[3rd Person] [PL] [PST]_
  (‘Yesterday (I/they) spoke’)
  b. Mañana hablará_[3rd Person] [SG] [FUT]_/hablarán_[3rd Person] [PL] [FUT]_
  (‘Tomorrow (he, she, it/they) will speak’)

Compared with Spanish and even inflectionally-restricted English verbal agreement, Chinese adopts few visible grammatical or inflectional morphology to indicate gender, case, or number, except for the controversial visible plural marker *mén* on pronouns or nouns (but not verbs). Moreover, common nouns are not compulsorily inflected for person and number in Chinese. In this case, grammatical agreement is expressed *via* independent function words, word order, or *via* context. The consequence of lacking explicit agreement morphology on Chinese verbs is revealed in language comprehension and processing (*cf.* Li, 2006). Chinese syntax has been claimed not to require (explicit) SV agreement, and seemingly any nominal subject can take any verb form (*cf.* Chen et al., 2007). Examples are listed in (7–8).

7. 我_[1st Person] [SG]_/你_[2nd Person] [SG]_/他_[3rd Person] [SG] [M]_/她_[3rd Person] [SG] [F]_/它_[3rd Person] [SG] [NH]_ 说.
  wŏ/nĭ/ tā (singular, male)/ tā (singular, female)/tā (singular, non-human) shuō
    I/you/he/she/it say
    ‘I/you say; he/she/it says.’
8. 我们_[1st Person] [PL]_/你们_[2nd Person] [PL]_/他们_[3rd Person] [PL] [M]_/她们_[3rd Person] [PL] [F]_/它们_[3rd Person] [PL] [NH]_ 说.
  wŏ mén /nĭ mén/tā mén (male)/ tā mén (female)/tā mén (non-human) shuō
    We/you/ they / they/they say
    ‘We/they say.’

For adverb-verb TC, the situation turns out to be different. Even if verbs do not bear explicit inflections for [Person] and [Number], they usually occur with particles that denote aspectual and modal information, and they concord with adverbs, which are canonically located before the verb. Examples are provided in (9–10).

9. a. **昨天**_[PST]_ 他 买 **了**_[PST]_ 一 本 有趣的 书。
  zuótiān tā mǎi le yī běn yoˇuqùde shū.
    yesterday he buy asp. one classifier interesting book
    ‘Yesterday, he bought an interesting book.’
  b. *昨天_[PST]_ 他 *将_[FUT]_ 买 一 本 有趣的 书。
    zuótiān tā jiāng mǎi yī běn yoˇuqùde shū
    yesterday he will buy one classifier interesting book
    ‘Yesterday, he would buy an interesting book.’
10. a. **在 不久的 将来**_[FUT]_， 她 **会**_[FUT]_ 解决 这个 问题。
  zài bùjiuˇde jiānglái, tā huì jiějué zhège wèntí
    in not long future she will solve this problem
    ‘In near future, she will solve this problem.’
    b. *在 不久的 将来_[FUT]_， 她 解决 *过_[PST]_ 这个 问题。
    zài bùjiuˇde jiānglái, tā jiějué guò zhège wèntí
    in not long future she solve past this problem
    ‘*In near future, she solved this problem.’

In (9a), the time adverbial (*zuótiān* ‘yesterday’) correctly concords with the particle (*le* ‘past aspectual marker’), while in (9b) the same adverb mismatches the futural modal verb (*jiāng* ‘will’). The same is true for (10). The adverbial of time (*bùjiuˇde jiānglái* ‘in near future’) in (10a) concords with the modal verb (*huì* ‘will/be likely to’), but the adverb in (10b) does not concord with the past aspectual marker (*guò* ‘past aspectual marker’), which is attached after the main verb (*jiějué* ‘solve’). The sentences (9b) and (10b) thus lead to ungrammaticality.

To sum up, differing from a morphologically rich language such as Spanish, Chinese does not resort to explicit inflectional morphology to mark person, gender, case, or number (in most situations). Moreover, unlike Spanish where agreement and tense features are expressed through morphological suffixes on the verb, Chinese temporal information is expressed through particles that express aspectual/modal information. Therefore, how SV agreement and adverb-verb TC are acquired by native Chinese speakers learning Spanish as an L2 is a pivotal question. The general prediction is that native Chinese speakers may show delays in the acquisition of verbal inflection in morphologically rich languages such as Spanish.

## 4. The current study

The current study aims to test Chinese Spanish L2ers’ grammatical knowledge of SV agreement and adverb-verb TC. In particular, this study investigates the extent to which L2ers use native-like processing mechanisms during L2 self-paced reading, thus providing a tentative cross-linguistic verification of the mechanism(s) underlying the processing of two relations. Since grammaticality judgment task data are reliable measures of linguistic knowledge (linguistic competence; Mandell, 1999), a grammaticality judgment task is used to check to what extent the participants master the relevant morphosyntactic knowledge.

Besides, the self-paced grammaticality judgment task is frequently used in the field of L2 acquisition (see Marsden et al., 2018 for a recent summary). This task allows researchers to observe the incremental processing of sentences, which is suggestive of specific mechanisms employed by L2ers for acquiring a certain language (Renaud, 2014), even without the examination of different proficiency groups. Specifically, we ask three questions related to the parsing mechanisms and the grammatical knowledge of advanced Chinese Spanish L2ers.^2^

### 4.1. Are advanced Chinese Spanish L2ers able to process L2 verbal inflection during online sentence processing given the unspecified morphological marking in Chinese?

Since Chinese and Spanish are maximally different from each other in terms of verbal morphology, Chinese Spanish L2ers may not be sensitive to inflectional errors, due to the L1 constraints on the acquisition of non-native morphology (e.g., Jiang, 2004; Roberts and Liszka, 2013). However, the advanced proficiency level of L2 Spanish may solve the acquisition challenges (e.g., Foote, 2011; Yao and Chen, 2017). Proficient Chinese Spanish L2ers may develop a more native-like degree of sensitivity to L2 verbal inflection. Moreover, Spanish verbs have a rich set of morphemes to express the target features and thus provide informative cues to the learners. This property of the L2 can lead to two different scenarios.

On the grounds of relative difficulty, the morphological richness of the L2 (Spanish) may make the acquisition of verbal morphology harder for speakers of an L1 with poor morphology (Chinese). This situation is particularly apparent for non-proficient learners who struggle with the detection/recognition of morphosyntactic cues due to cross-linguistic differences. Under this scenario, we would expect unskilled and non-proficient Chinese Spanish L2ers to have lower accuracy in grammaticality judgments and delayed detection of errors during self-paced reading, compared with native Spanish speakers. However, advanced Chinese Spanish L2ers (as in the current study) should be able to detect the grammatical errors as accurately as native Spanish speakers, and show differences in reading time at the critical words between grammatical and ungrammatical conditions.

On the grounds of cue validity, Spanish provides rich morphological information that can help the L2 learners of Spanish to shift their attention from semantic processing to morphosyntactic processing (*cf.* Kempe and MacWhinney, 1998). In general, Chinese is such a morphologically impoverished language that it mostly resorts to the context to convey the abstract grammatical information such as number or gender. Native Chinese speakers could thus primarily rely on grammatically semantic or pragmatic cues to process SV agreement and adverb-verb TC relations. However, when learning a morphologically rich language such as Spanish, they can utilize those rich morphological cues on Spanish verbs to realize the morphosyntactic mapping and extract the morphosyntactic properties of Spanish verbal inflection. Under this hypothesis, we would expect Chinese Spanish learners to be as capable as native Spanish speakers in using morphosyntactic processing to comprehend SV agreement and adverb-verb TC relations, rather than relying on semantic processing.

In sum, we hypothesize that native Spanish speakers and advanced Spanish L2ers in our study should correctly reject ungrammatical conditions (mismatched [Person], [Number], or [Tense]) and the mismatched effects should also be revealed by RT data (either at the target verb, or on the following words).

### 4.2. Does the processing of Spanish verbal inflection vary according to the types of relation (agreement vs. concord) and/or the distances between the subject/adverb and the verb (local vs. distal)?

As mentioned previously, tense errors were detected earlier than SV agreement errors when Chinese English L2ers read English sentences (Yao and Chen, 2017). Note that compared with the tense marker-ed with only [Past], the English agreement marker-s is more complex since it is associated with phi-features such as [3rd Person] and [Singular], and [-Past] Tense feature (Lardiere, 1998). Therefore, it is unclear whether the processing differences could stem from the different features bundled on the related markers or from different cognitive mechanisms at play during the processing of different types of verbs. Moreover, it is unclear whether this conundrum related to the processing of English verbs would also occur during the processing of Spanish SV agreement and adverb-verb TC for advanced Chinese Spanish learners.

The current study focuses on whether different relations and linear distance influence the parsing of the Spanish verbal inflection. Each change in feature values of [Person], [Number] and [Tense] produces an independent verb form in Spanish. As a consequence, the influence of the feature bundle can be well-controlled. If advanced Chinese Spanish L2ers can process agreement and concord in a native-like manner, like other L2ers whose L1s are morphologically comparatively poor (e.g., English Spanish L2ers in Biondo and Mancini, 2021), they should have higher accuracy in judging subject-verb relations than in judging adverb-verb relations, and detect agreement violations earlier (at the target verb) than concord violations (at the word following the target verb). Moreover, based on the eye-movement findings of native Spanish speakers (Biondo et al., 2018), the processing differences between SV agreement and adverb-verb TC could be more likely to arise in the local configuration than in the distal configuration. Finally, due to the cross-linguistic similarities and differences, Chinese Spanish L2ers are more likely to have difficulties with the acquisition of SV agreement than with the acquisition of adverb-verb TC. Adverb-verb TC violations should thus be detected with higher accuracy and earlier in the sentences compared to SV agreement violations for Chinese Spanish L2ers.

### 4.3. Does the specific type of phi-feature influence Chinese Spanish L2ers’ ability to process SV agreement?

In Chinese, there is no inflectional morphology on the verb. When a human noun is assigned with the plural value, the plural marker *mén* is optionally present. If the optional use of number morphology on the noun and the absence of verb (number/person) morphology in the L1 has a strong influence on L2 morphological processing, we expect advanced Chinese Spanish L2ers not to be sensitive to number violations but highly sensitive to person violations during self-paced reading, in line with previous studies (e.g., Jiang, 2004; Shibuya and Wakabayashi, 2008).

If late learners can overcome L2 acquisition difficulties when their L2 proficiency increases, we expect advanced Chinese Spanish L2ers to show a native-like sensitivity to both person and number violations. To be specific, they should show bigger effects (longer RT) for person violations than for number violations in the local configuration and equivalent sensitivity to these two types of violations in the distal configuration, in line with what was found by native Spanish speakers (Biondo et al., 2018).

## 5. Materials and methods

### 5.1. Participants

The participants consisted of 21 native Spanish speakers (10 females, 11 males; mean age: 19.4; range:17–22) and 47 Chinese Spanish L2ers (42 females, 5 males; mean age: 21.3; range: 20–22). The native Spanish speakers were undergraduate students at either Pompeu Fabra University (*n* = 19) or Jaume I University (*n* = 2). It is noted that Pompeu Fabra University is located in a Catalan–Spanish bilingual context.^3^ Although Spanish and Catalan show subtle syntactic differences (e.g., the Spanish preterite is more complex than the Catalan preterite), they are both morphologically rich languages and recruit the same Agree mechanism. Therefore, this should not preclude us from answering the research questions. The L2 learners of Spanish (with comparatively low English proficiency^4^) were undergraduate students at Soochow University. Before the formal experiment, only Chinese Spanish L2ers (not native Spanish speakers) were asked to complete a short proficiency test, which was taken from Child (2013) and had been widely used in L2 acquisition research (e.g., Montrul et al., 2008). The test included a multiple choice vocabulary section from a Modern Language Association test (30 items) and a cloze section from the advanced Diplomas de Espãnol como Lengua Extranjera (20 items). Among the 47 learners, 31 of them had an accuracy above 75%, a cutoff point for being considered as an advanced learner according to Foote (2011). Only these advanced Chinese Spanish L2ers (29 females, 2 males; mean age: 21; range: 20–22) were selected to participate in the following self-paced reading experiment. Participant characteristics are summarized in Table 1.

**Table 1:** Participant characteristics of the self-paced reading experiment.

	Native Spanish speaker	Chinese Spanish L2er
Total number	21	31
Mean age (range)	19.4 (17–22)	21 (19–22)
Female:Male	11:10	29:2
Mean Spanish proficiency	–	84.72%
Years of exposure (range)	–	2.9 (1.3–4.3)
Onset age (range)	–	18.1 (17–19)
Frequency of use of Spanish (range)	–	3.54 (3–4.4)*

^*^The frequency of use of Spanish was evaluated through their Spanish class hour (h/week) since our Chinese Spanish L2ers were full-time undergraduate students majoring in Spanish Language and Literature.

### 5.2. Materials

The experimental material consisted of 100 item sets of Spanish sentences, all of which were taken from our previous L1 studies (Biondo, 2017; Biondo et al., 2018) and had been proofread and validated by native Spanish speakers. Each sentence contained an animate noun/determiner phrase in the subject position, a deictic temporal adverb (e.g., *mañana/ayer por la tarde* ‘tomorrow/yesterday noon’), and a lexical verb followed by a direct/indirect object DP. Past-marking adverbs were used in the half of the experimental sentences, while future-marking adverbs were chosen in the other half.

As shown in Table 2, 10 versions of each experimental sentence were created. We manipulated both subject-verb and adverb-verb relations (relation type factor). Note that the stimuli used for subject-verb manipulations all contained third person singular subjects (e.g., *el viajero cansado* ‘the tired traveler’), while third person plural subjects (e.g., *los viajeros cansados* ‘the tired travelers’) were used for adverb-verb tense manipulations.^5^ Within each relation, we manipulated [Number], [Person], and [Tense] encoded in verbal morphology to form grammatical and ungrammatical sentences (grammaticality factor). In addition, the linear distance between the subject/adverb and the verb was also manipulated to create local and distal relations (configuration factor).

**Table 2:** A sample of the material (SV agreement: control 1, number and person; adverb-verb TC: control 2, tense) in the two configurations (subject/adverb-verb relation: local, distal).

	Local	Distal
Control 1	*Mañana al mediodía* el viajero cansado_[3rd Person] [SG]_ **regresará**_[3rd Person] [SG]_ a casa con mucho equipaje. (*Tomorrow at noon* the tired traveler_[3rd Person] [SG]_ will **go**_[3rd Person] [SG]_ back home with a lot of bags)	El viajero cansado_[3rd Person] [SG]_ *mañana al mediodía* **regresará**_[3rd Person] [SG]_ a casa con mucho equipaje. (The tired traveler_[3rd Person] [SG]_ *tomorrow at noon* will go_[3rd Person] [SG]_ back home with a lot of bags)
Number	*Mañana al mediodía el viajero cansado_[3rd Person] [SG]_ **regresarán**_[3rd Person] [PL]_ a casa con mucho equipaje. (*Tomorrow at noon* the tired traveler_[3rd Person] [SG]_ will go_[3rd Person] [PL]_ back home with a lot of bags)	*El viajero cansado_[3rd Person] [SG]_ *mañana al mediodía* **regresarán**_[3rd Person] [PL]_ a casa con mucho equipaje. (The tired traveler_[3rd Person] [SG]_ *tomorrow at noon* will go_[3rd Person] [PL]_ back home with a lot of bags)
Person	*Mañana al mediodía el viajero cansado_[3rd Person] [SG]_ **regresarás**_[2nd Person] [SG]_ a casa con mucho equipaje. (*Tomorrow at noon* the tired traveler_[3rd Person] [SG]_ will go_[2nd Person] [SG]_ back home with a lot of bags)	*El viajero cansado_[3rd Person] [SG]_ *mañana al mediodía* **regresarás**_[2nd Person] [SG]_ a casa con mucho equipaje. (The tired traveler_[3rd Person] [SG]_ *tomorrow at noon* will go_[2nd Person] [SG]_ back home with a lot of bags)
Control 2	Los viajeros cansados*mañana al mediodía*_[FUT]_ **regresarán**_[FUT]_ a casa con mucho equipaje. (The tired travelers *tomorrow at noon*_[FUT]_ will **go**_[FUT]_ back home with a lot of bags)	*Mañana al mediodía*_[FUT]_ los viajeros cansados**regresarán**_[FUT]_ a casa con mucho equipaje. (*Tomorrow at noon*_[FUT]_ the tired travelers will **go**_[FUT]_ back home with a lot of bags)
Tense	*Los viajeros cansados *mañana al mediodía*_[FUT]_ **regresaron**_[PST]_ a casa con mucho equipaje. (The tired travelers *tomorrow at noon*_[FUT]_ **went**_[PST]_ back home with a lot of bags)	*Mañana al mediodía_[FUT]_ los viajeros cansados **regresaron**_PST_ a casa con mucho equipaje. (*Tomorrow at noon*_[FUT]_ the tired travelers **went**_[PST]_ back home with a lot of bags)

All the critical experimental sentences were distributed across 10 lists following a Latin square design,^6^ such that each condition contained 10 experimental sentences and participants only read one experimental sentence from the same item set. Moreover, sixty filler sentences were added to each list (20 ungrammatical sentences containing number violations with plural subjects, and 40 grammatical sentences containing unagreement patterns and historical present tense), in order to balance the proportion of grammatical and ungrammatical sentences and vary the type of agreement and tense manipulation, as in Biondo et al. (2018).

### 5.3. Task and procedure

All subjects gave their informed consent for inclusion and completed a background questionnaire before they participated in the study. The sentences were presented word by word in black letters (18 point Courier New font) against a grey background,^7^ by using the moving-window paradigm.

Each trial began with the sentence masked as many dashes as the number of letters per word (e.g., “The” is masked by three dashes “---”), with the words separated by spaces. The participant, seated in front of a computer, was asked to press the spacebar to read each following word. As each word appeared, the preceding word was re-masked. The period appeared together with the final word. Reaction times (RTs) were recorded from the time each word appeared on the screen until the spacebar was pressed to read the next word. After the offset of the sentence, a blank screen appeared for 500 milliseconds (ms), followed by the words *Bien* ‘good’ and *Mal* ‘bad’. Participants were instructed to read at a natural pace and to judge whether the sentences presented were possible sentences in Spanish. The response was given by pressing one of two lateral keys (i.e., “J” and “F,” corresponding to *Bien* and *Mal*) on a keyboard. The next trial began after 500 ms. The sentences were presented in a pseudorandom order, such that no more than three items of the same condition were presented one after the other. The experimental session was preceded by 16 practice trials to familiarize the participant with the procedure. Experimental trials were presented in four blocks (40 items per block) of about 7 min each, separated by brief rest breaks.

## 6. Data analyses and results

Statistical analyses were conducted on the accuracy of grammaticality judgment and logarithmic RTs of the inflected verb and the three following words (verb +1, verb +2, and verb +3). Any participant whose grammaticality judgment error rate was higher than 30% was excluded from RT data analyses. Two Chinese Spanish L2ers were excluded for this reason, leaving the total number of L2ers at 29. Moreover, RTs that were 2 standard deviations away from an individual’s mean (Roberts and Liszka, 2013), or shorter than low cutoff set at 100 ms were discarded. These procedures, along with excluding the incorrect responses, affected 7.80% of the native Spanish speaker and 15.08% of the Chinese Spanish L2er data. One might suggest using residual RTs as the dependent variable to control individual reading speed rates. However, an inspection of the RT distribution revealed a skewed distribution which cannot be handled by using residual RTs. Thus, a logarithmic transformation was chosen to normalize the distribution.

For each analysis, we reported the estimated regression coefficient (Estimate), standard error (SE) and *t*/Wald’s *z* values for only significant effects and comparisons.

### 6.1. Comparison between adverb-verb and subject-verb violations

To address the Research Questions 1 and 2, the accuracy and the logarithmic RT data were analyzed with one between-participants factor (language group: Chinese Spanish L2ers and native Spanish speakers) and three within-participants factors (grammaticality: grammatical and ungrammatical; relation type: subject-verb and adverb-verb; configuration: local and distal). The analyses were carried out fitting linear (LMM, for logarithmic RT) and logistic (GLMM, for accuracy) mixed effect models by using the lme4 package (Version 1.1-26; Bates et al., 2015b) in R (Version 4.0.3; R Core Team, 2021) and RStudio (Version 1.4.1103; R Studio Team, 2021). In each model, we included language group, relation type, grammaticality, configuration and interactions involving grammaticality (i.e., grammaticality × relation type, grammaticality × language group, grammaticality × configuration, grammaticality × relation type × language group, grammaticality × relation type × configuration, grammaticality × configuration × language group, grammaticality × relation type × configuration × language group) as fixed effects. As for the random intercepts and slopes, we started with the maximal random-effect structure and then reduced the degree of complexity by choosing the best-fitting parsimonious model (Bates et al., 2015a). Likelihood ratio tests (calculated by the R package *lmerTest*, *cf.* Kuznetsova et al., 2017) were adopted to evaluate whether the exclusion of a random-effect parameter provided a better fit of the model. More complex models were disregarded only if the value of *p* for the significance of the difference between two models was below 0.05.

#### 6.1.1. Accuracy

**Figure 1:**
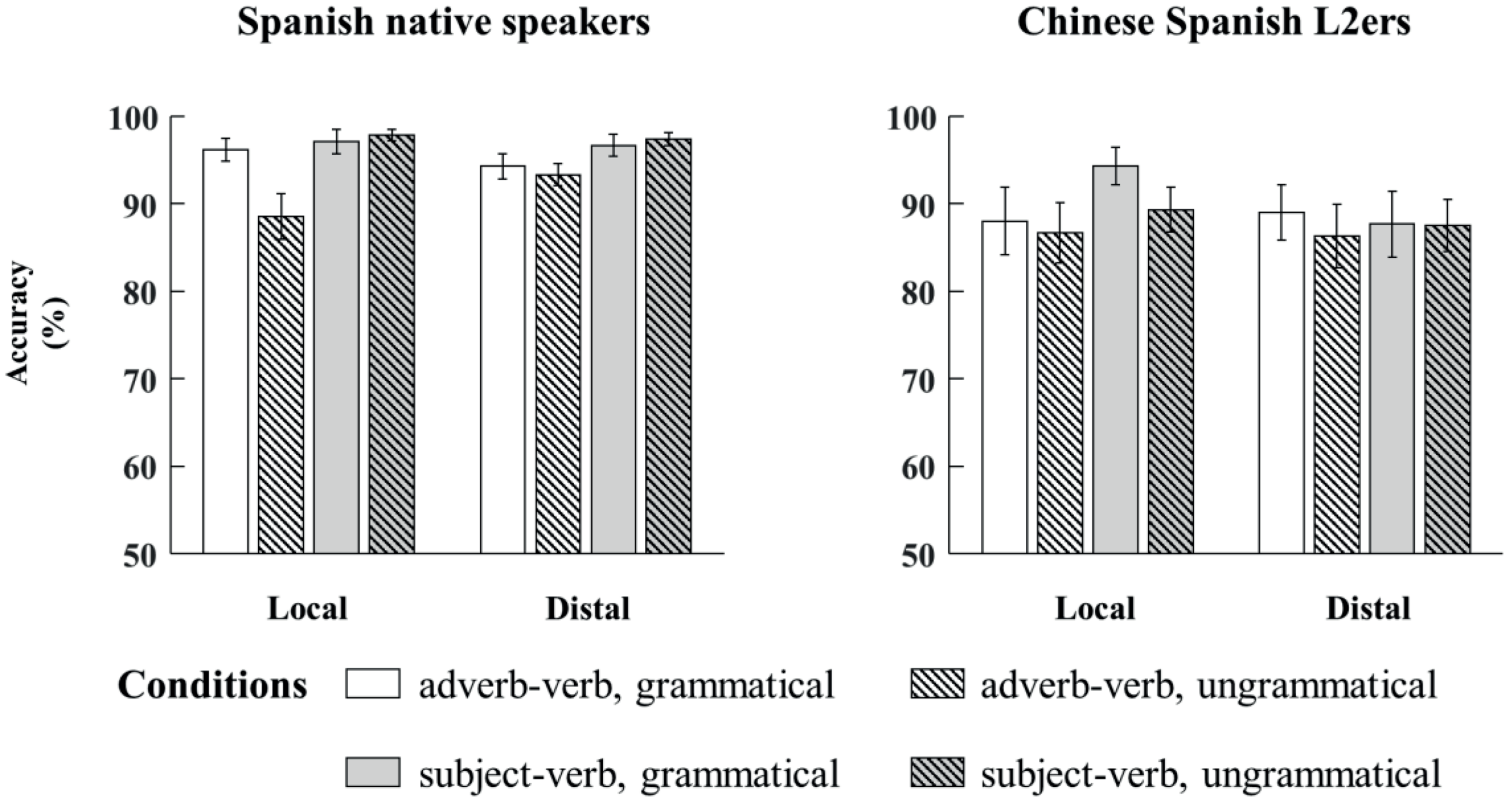
Bar plots of mean accuracy (and by-subject standard errors) to adverb-verb TC and SV agreement in the two configurations from native Spanish speakers and Chinese Spanish L2ers.

Statistical analyses did not show any main effect of grammaticality or interactions with grammaticality (*p*s > 0.05). We found main effects of language group (Estimate = −0.43, SE = 0.13, *z* = −3.34, *p* < 0.001), relation type (Estimate = −0.26, SE = 0.09, *t* = −2.94, *p* < 0.01), and configuration (Estimate = −0.12, SE = 0.06, *t* = −2.03, *p* < 0.05). The Chinese Spanish L2ers were less accurate than the native Spanish speakers, and the same for the adverb-verb relation compared to the subject-verb relation and for the distal configuration compared to the local configuration (see Figure 1).

#### 6.1.2. Reading times

**Figure 2:**
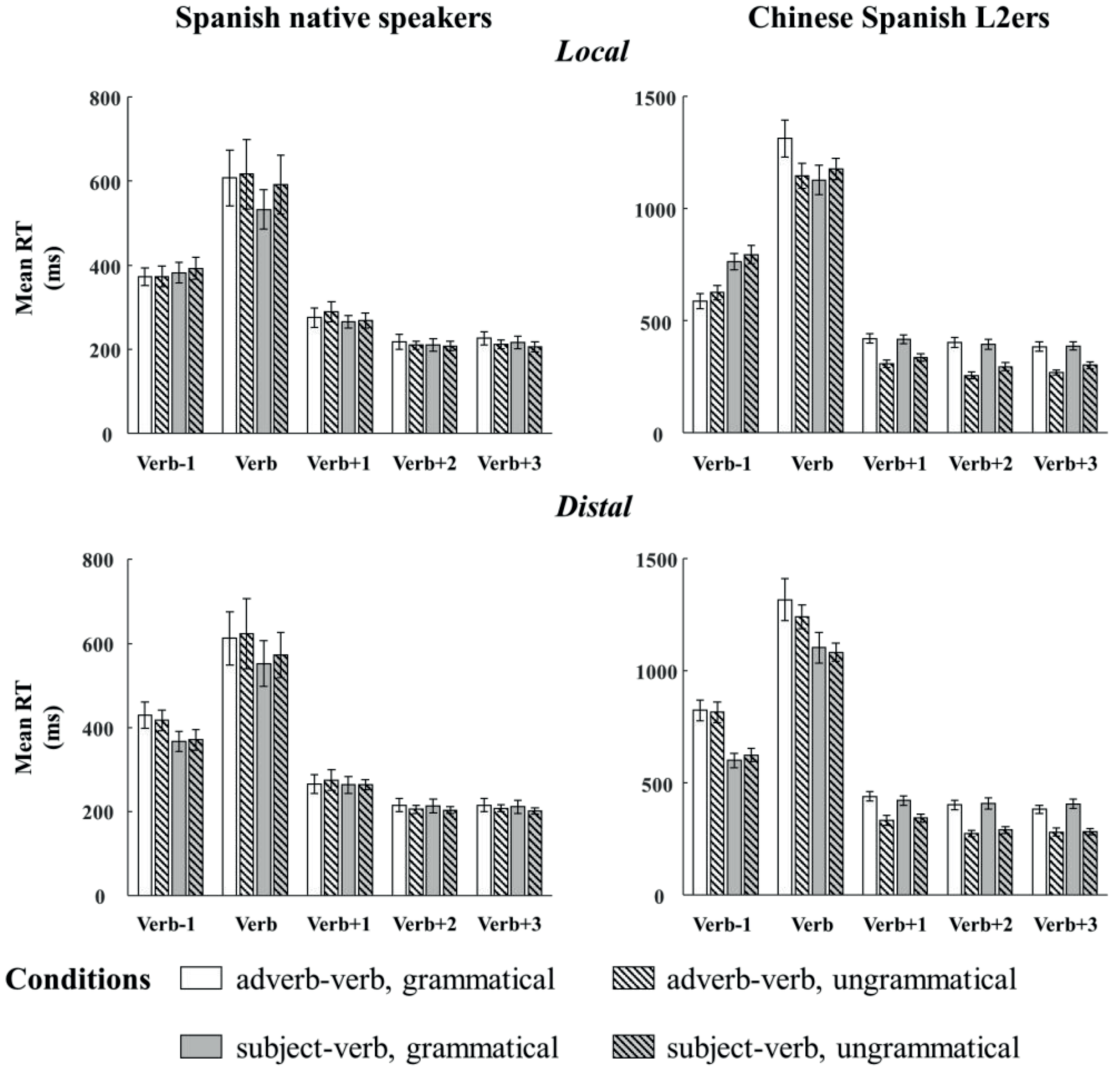
Bar plots of mean RTs (and by-subject standard errors) to adverb-verb TC and SV agreement in the two configurations from native Spanish speakers and Chinese Spanish L2ers. Mean RTs were divided into five regions: the target verb, one preceding word, and three following words.

Figure 2 and Table 3 respectively present the mean RTs and the results of the linear mixed effect models for each region of interest (Verb, Verb +1, Verb +2, and Verb +3) and for adverb-verb and subject-verb violations. Mean RTs are given for descriptive purpose only. As shown in Table 3, the main effects of language group were consistently significant across all regions, indicating that Chinese Spanish L2ers read the verb and its three following words slower than native Spanish speakers.

**Table 3:** Summary of the linear mixed effect models for the comparison between adverb-verb and subject-verb violations.

Predictor fixed effects	Verb				Verb +1				Verb +2				Verb +3			
	Estimate	SE	*t*	Value of *p*	Estimate	SE	*t*	Value of *p*	Estimate	SE	*t*	Value of *p*	Estimate	SE	*t*	Value of *p*
Intercept	6.568	0.045	145.573	<0.001***	5.656	0.033	173.762	<0.001***	5.501	0.034	162.872	<0.001***	5.509	0.032	172.072	<0.001***
L	0.372	0.046	8.147	<0.001***	0.143	0.032	4.497	<0.001***	0.196	0.034	5.850	<0.001***	0.196	0.031	6.235	<0.001***
G	0.002	0.013	0.184	0.855	−0.070	0.012	−6.086	<0.001***	−0.102	0.014	−7.307	<0.001***	−0.095	0.011	−8.975	<0.001***
R	0.044	0.007	6.313	<0.001***	−0.005	0.006	−0.906	0.365	−0.010	0.005	−2.161	<0.05*	−0.006	0.005	−1.258	0.209
C	−0.006	0.007	−0.803	0.422	0.007	0.006	1.136	0.256	0.009	0.005	1.833	0.067	0.001	0.005	0.193	0.847
C × G	−0.007	0.007	−0.970	0.332	0.000	0.006	0.032	0.974	0.000	0.005	0.049	0.961	−0.000	0.005	−0.012	0.991
R × G	−0.023	0.007	−3.213	0.001**	−0.005	0.006	−0.928	0.354	−0.012	0.005	−2.424	<0.05*	−0.002	0.006	−0.394	0.695
L × G	−0.013	0.013	−0.989	0.328	−0.073	0.011	−6.319	<0.001***	−0.089	0.014	−6.206	<0.001***	−0.073	0.011	−6.910	<0.001***
R × C × G	0.006	0.007	0.878	0.380	0.000	0.008	0.012	0.991	0.009	0.005	1.793	0.073	0.007	0.005	1.553	0.120
L × C × G	−0.000	0.007	−0.012	0.990	0.001	0.006	0.106	0.915	0.003	0.005	0.704	0.482	−0.005	0.005	−1.184	0.237
L × R × G	−0.004	0.007	−0.614	0.539	−0.011	0.006	−1.948	0.052	−0.012	0.005	−2.551	<0.05*	−0.005	0.006	−0.791	0.433
L × R × C × G	0.013	0.007	1.947	0.052	−0.002	0.007	−0.290	0.773	0.006	0.005	1.316	0.188	0.004	0.005	0.948	0.343

G, Grammaticality; R, Relation type; C, Configuration; L, Language group. *p < 0.05; **p < 0.01; ***p < 0.001.

At the target verb region, there were also a main effect of relation type and a two-way interaction (relation type × grammaticality). Follow-up analyses showed that the ungrammatical verbs were read marginally significantly slower than the grammatical verbs in the subject-verb relations (Estimate = 0.02, SE = 0.01, *t* = 2.01, *p* = 0.05), while no difference was found in the adverb-verb relations (Estimate = −0.03, SE = 0.02, *t* = −1.69, *p* = 0.10). Although the descriptive results (see Figure 2) showed different reading patterns in the adverb-verb conditions for the native Spanish speakers (grammatical: 610.60 ms, ungrammatical: 622.50 ms) and the Chinese Spanish L2ers (grammatical: 1315.10 ms, ungrammatical: 1183.67 ms), the interaction (language group × relation type × grammaticality) was not significant. However, the absence of a significant effect should be carefully interpreted as it cannot be seen as a conclusive result due to the relatively small sample size in the current study.

At the following regions, the main effect of grammaticality and the two-way interaction (language group × grammaticality) were consistently significant. Besides, at the second word following the target, the main effect of relation and the interaction (language group × relation type × grammaticality) were also significant. These effects were caused by the fact that the Chinese Spanish L2ers spent less time in reading these words in the ungrammatical condition than in the grammatical condition (Verb +1: Estimate = −0.14, SE = 0.02, *t* = −7.42, *p* < 0.001; Verb +2 (adverb-verb): Estimate = −0.21, SE = 0.02, *t* = −10.18, *p* < 0.001; Verb +2 (subject-verb): Estimate = −0.17, SE = 0.03, *t* = −6.67, *p* < 0.001; Verb +3: Estimate = −0.17, SE = 0.02, *t* = −9.69, *p* < 0.001), but the native Spanish speakers only showed the speed-up effect at the third word following the target (Verb +1: Estimate = 0.003, SE = 0.01, *t* = 0.33, *p* = 0.74; Verb +2 (adverb-verb): Estimate = −0.01, SE = 0.01, *t* = −0.67, *p* = 0.51; Verb +2 (subject-verb): Estimate = −0.01, SE = 0.01, *t* = −0.87, *p* = 0.39; Verb +3: Estimate = −0.02, SE = 0.01, *t* = −2.69, *p* < 0.05).

### 6.2. Comparison between number and person violations

To address the research question 3, we focused on the subject-verb relations and conducted separate analyses with one between-participants factor (language group: Chinese Spanish L2ers and native Spanish speakers) and two within-participants factors (grammaticality: person, number and control; configuration: local and distal). The grammaticality factor was coded by using repeated contrasts: the first comparison was conducted between grammatical and ungrammatical SV number agreement (number vs. control) and the second comparison was conducted between grammatical and ungrammatical SV person agreement (person vs. control). Accuracy and logarithmic RT data were modeled by using language group, configuration, grammaticality, and interactions involving grammaticality (i.e., grammaticality × language group, grammaticality × configuration, and grammaticality × configuration × language group for SV person agreement and for SV number agreement respectively) as fixed effects. The random-effect structure was chosen with a similar procedure mentioned in the preceding subsection 6.1.

#### 6.2.1. Accuracy

**Figure 3:**
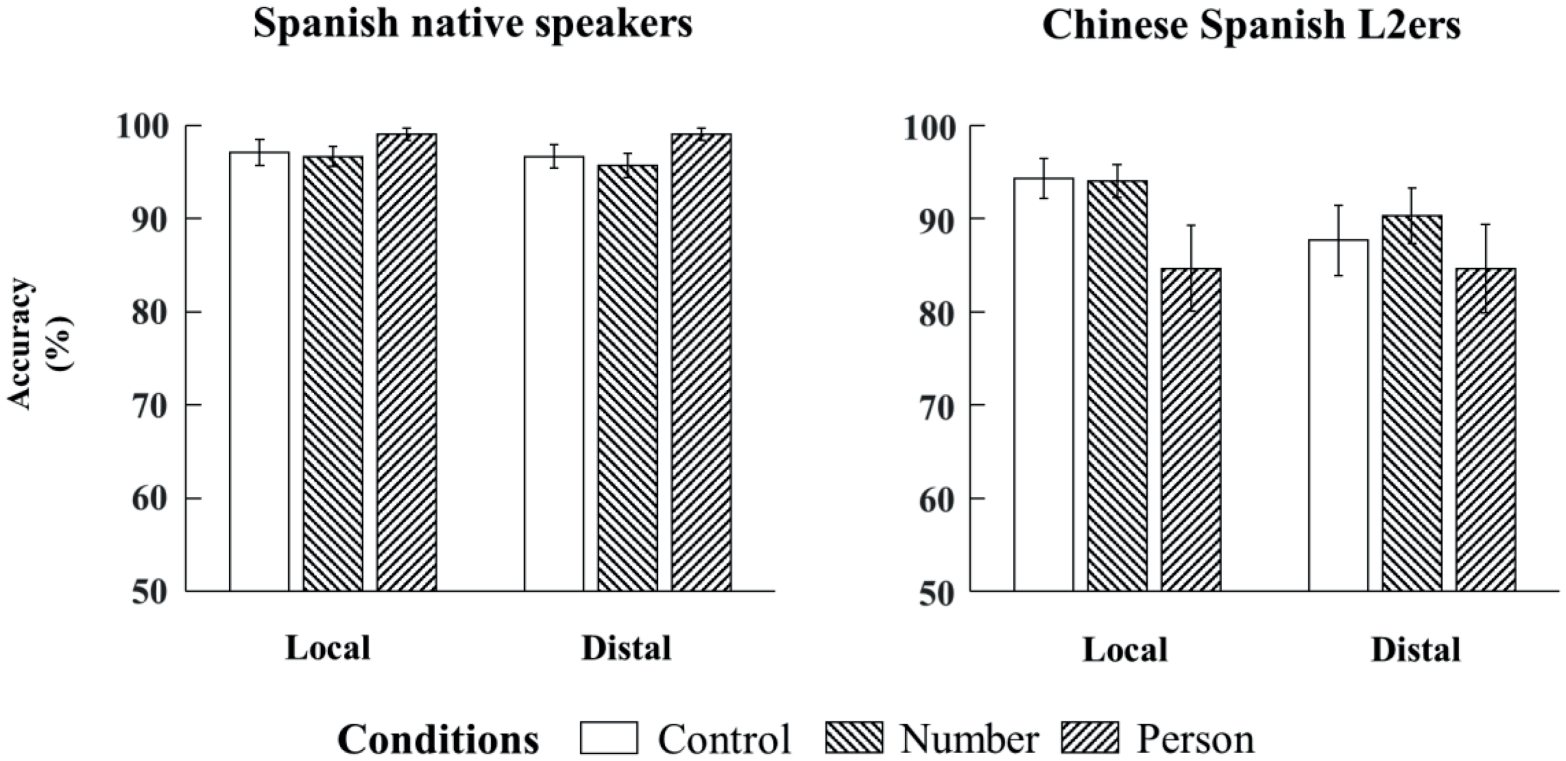
Bar plots of mean accuracy (and by-subject standard errors) to SV agreement in the two configurations from native Spanish speakers and Chinese Spanish L2ers.

Results showed main effects of language group (Estimate = −0.82, SE = 0.20, *z* = −4.10, *p* < 0.001) and configuration (Estimate = −0.24, SE = 0.09, *z* = −2.81, *p* < 0.05). That is, the Chinese Spanish L2ers had lower accuracy than the native Spanish speakers, and the same for the distal configuration compared to the local configuration (see Figure 3). Moreover, there was a two-way interaction (language group × grammaticality) for SV person agreement (Estimate = −0.99, SE = 0.47, *z* = −2.10, *p* < 0.05). Follow-up analyses showed that the native Spanish speakers judged the ungrammatical sentences more accurately than the grammatical sentences (Estimate = 0.65, SE = 0.30, *z* = 2.14, *p* < 0.05), while the Chinese Spanish L2ers did not show this difference (Estimate = −0.17, SE = 0.27, *z* = −0.63, *p* = 0.53).

#### 6.2.2. Reading times

**Figure 4:**
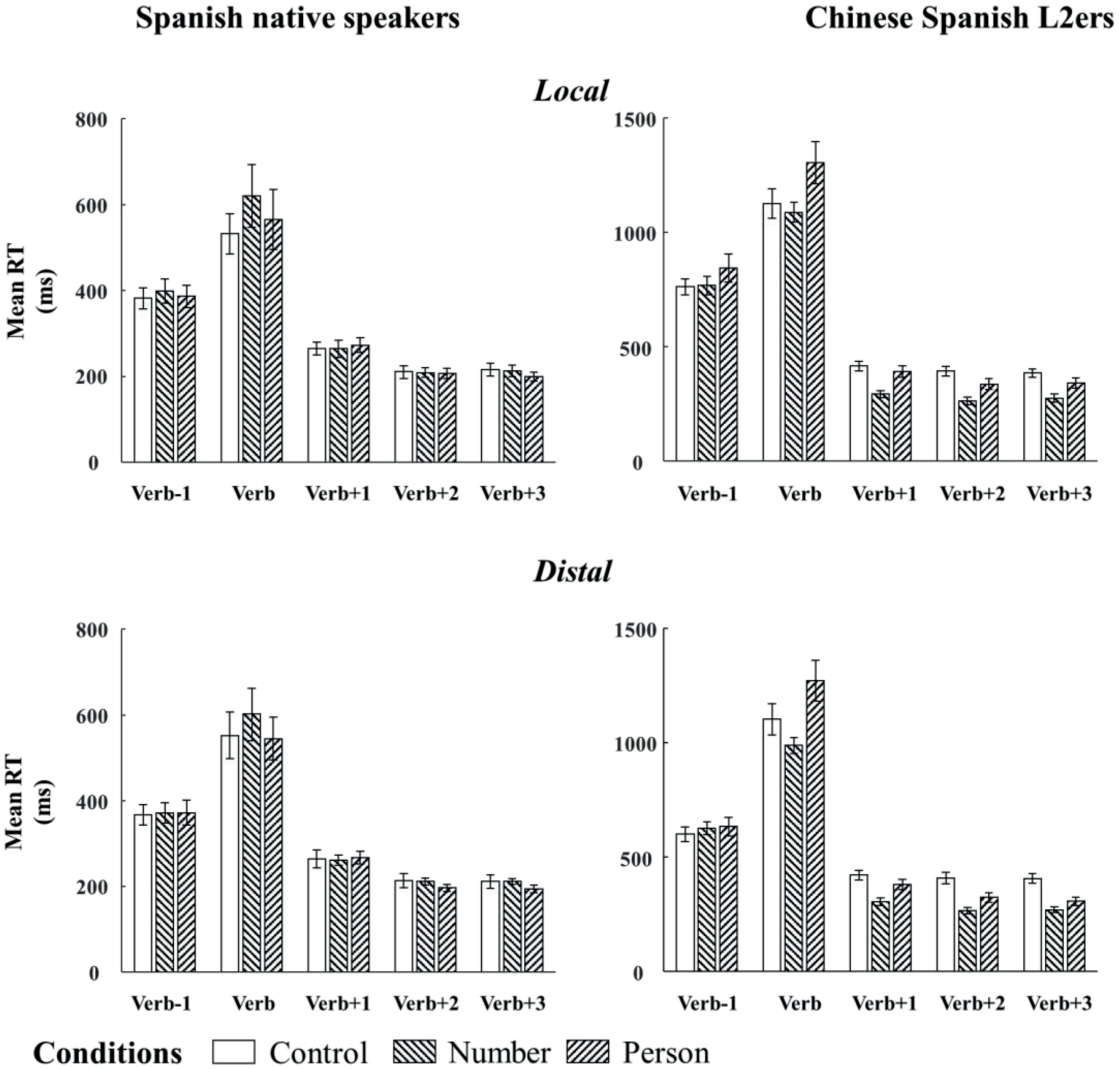
Bar plots of mean RTs (and by-subject standard errors) to SV agreement in the two configurations from native Spanish speakers and Chinese Spanish L2ers. Mean RTs were divided into five regions: the target verb, one preceding word, and three following words.

Figure 4 and Table 4 respectively present the mean RTs and the results of the linear mixed effect model analyses for each region of interest (Verb, Verb +1, Verb +2, and Verb +3) and for number and person violations. Similar to the Tables 3, 4 also reveals a significant main effect of language group across all regions, indicating that the Chinese Spanish L2ers read the words more slowly than the native Spanish speakers.

**Table 4:** Summary of the linear mixed effect models for the comparison between number and person violations.

Predictor fixed effects	Verb				Verb +1				Verb +2				Verb +3			
	Estimate	SE	*t*	Value of *p*	Estimate	SE	*t*	Value of *p*	Estimate	SE	*t*	Value of *p*	Estimate	SE	*t*	Value of *p*
Intercept	6.535	0.044	148.531	<0.001***	5.641	0.032	175.492	<0.001***	5.483	0.034	159.288	<0.001***	5.484	0.033	167.541	<0.001***
L	0.361	0.045	8.083	<0.001***	0.131	0.031	4.225	<0.001***	0.186	0.034	5.491	<0.001***	0.188	0.032	5.835	<0.001***
G																
nb vs. ct	0.037	0.021	1.797	0.072	−0.187	0.017	−10.896	<0.001***	−0.206	0.029	−7.083	<0.001***	−0.198	0.019	−10.168	<0.001***
ps vs. ct	0.074	0.025	2.97	0.004**	−0.059	0.022	−2.698	0.009**	−0.133	0.029	−4.574	<0.001***	−0.156	0.019	−8.023	<0.001***
C	−0.024	0.008	−2.838	0.005**	0.007	0.007	0.939	0.348	0.003	0.006	0.446	0.656	−0.005	0.006	−0.918	0.359
C × G																
C × G (nb vs. ct)	−0.024	0.021	−1.151	0.25	0.004	0.017	0.256	0.798	−0.005	0.014	−0.343	0.732	0.001	0.014	0.055	0.956
C × G (ps vs. ct)	−0.016	0.021	−0.748	0.454	−0.007	0.017	−0.425	0.671	−0.026	0.014	−1.782	0.075	−0.023	0.017	−1.365	0.178
L × G																
L × G (nb vs. ct)	−0.069	0.021	−3.322	<0.001***	−0.17	0.017	−9.896	<0.001***	−0.212	0.029	−7.27	<0.001***	−0.194	0.019	−9.946	<0.001***
L × G (ps vs. ct)	0.057	0.025	2.279	0.026*	−0.074	0.02	−3.718	<0.001***	−0.098	0.029	−3.361	0.002**	−0.091	0.019	−4.682	<0.001***
L × C × G																
L × C × G (nb vs. ct)	−0.01	0.021	−0.49	0.624	−0.004	0.017	−0.259	0.795	−0.01	0.014	−0.712	0.476	−0.017	0.014	−1.234	0.217
L × C × G (ps vs. ct)	−0.014	0.021	−0.649	0.517	−0.002	0.017	−0.124	0.901	−0.011	0.014	−0.748	0.454	−0.028	0.017	−1.63	0.109

G, Grammaticality; ps, person; nb, number; ct, control; C, Configuration; L, Language group. *p < 0.05; **p < 0.01; ***p < 0.001.

At the target verb region, there were a main effect of grammaticality and an interaction (language group × grammaticality). Follow-up analyses showed that the native Spanish speakers spent significantly more time in reading the ungrammatical verb than the grammatical verb when there was an SV number violation (ungrammatical: 610.97 ms, grammatical: 542.35 ms; Estimate = 0.10, SE = 0.03, *t* = 3.66, *p* < 0.001). Conversely, the SV person violation effect was not significant (ungrammatical: 554.84 ms, grammatical: 542.35 ms; Estimate = 0.02, SE = 0.03, *t* = 0.56, *p* = 0.58). The opposite pattern arose for the L2 group (see Figure 4): the Chinese Spanish L2ers spent significantly more time in reading the ungrammatical verb than the grammatical verb when there was an SV person violation (ungrammatical: 1275.84 ms, grammatical: 1120.81 ms; Estimate = 0.13, SE = 0.03, *t* = 4.12, *p* < 0.001), while the SV number violation effect was not significant (ungrammatical: 1038.76 ms, grammatical: 1120.81 ms; Estimate = −0.03, SE = 0.03, *t* = −0.97, *p* = 0.33). Finally, the main effect of configuration was significant, indicating that the verbs were read faster in the distal configuration than in the local configuration.

At the following regions, the main effect of grammaticality and the two-way interaction (language group × grammaticality) were consistently significant. Follow-up analyses showed that the Chinese Spanish L2ers spent less time in reading these words in the ungrammatical condition than in the grammatical condition both during SV number agreement (Verb +1: Estimate = −0.36, SE = 0.03, *t* = −13.77, *p* < 0.001; Verb +2: Estimate = −0.42, SE = 0.05, *t* = −8.77, *p* < 0.001; Verb +3: Estimate = −0.39, SE = 0.03, *t* = −12.23, *p* < 0.001), and SV person agreement (Verb +1: Estimate = −0.13, SE = 0.04, *t* = −3.76, *p* < 0.001; Verb +2: Estimate = −0.23, SE = 0.05, *t* = −4.71, *p* < 0.001; Verb +3: Estimate = −0.24, SE = 0.03, *t* = −7.14, *p* < 0.001). However, the native Spanish speakers only showed a speed-up effect at the second and the third word following the target during SV person agreement (Verb +1: Estimate = 0.02, SE = 0.02, *t* = 0.81, *p* = 0.42; Verb +2: Estimate = −0.03, SE = 0.01, *t* = −2.68, *p* < 0.01; Verb +3: Estimate = −0.06, SE = 0.01, *t* = −5.22, *p* < 0.001), but not during SV number agreement (Verb +1: Estimate = −0.02, SE = 0.02, *t* = −0.81, *p* = 0.42; Verb +2: Estimate = 0.01, SE = 0.02, *t* = 0.46, *p* = 0.65; Verb +3: Estimate = 0.003, SE = 0.01, *t* = 0.18, *p* = 0.85).

## 7. General discussion

In this article, we examined advanced Chinese Spanish L2ers’ and native Spanish speakers’ capacity of detecting Spanish agreement/concord violations by adopting the self-paced reading technique integrated with a grammaticality judgment task, primarily in order to address three research questions related to the processing mechanisms of advanced Chinese learners of L2 Spanish.

### 7.1. Chinese Spanish L2ers are able to process verbal inflection during online sentence processing

The first research question concerns the capacity of late Chinese Spanish L2ers to process verbal inflection. The overall results show that the L2 learners have a relatively slower self-paced reading speed and lower judgment accuracy compared with native Spanish speakers. Slower processing speeds during L2 reading compared to native reading is often found in L2 research (as one reviewer pointed out). The group differences in the grammaticality judgment task may reflect that the Chinese Spanish L2ers have not fully acquired Spanish verbal inflection. Moreover, the L2 learners only received short periods of classroom instruction (2.9 years, see Table 1) and barely had the opportunity to use the L2 outside of the classroom. Cross-linguistic differences (the lack of verbal inflection in their L1) could also have impacted the processing of L2 morphology and subsequently lead to less accurate grammaticality judgments.

It should be noted, however, that the score rates in the grammaticality judgment task were relatively high (88.60%), thus indicating that the L2 learners of our study had enough L2 knowledge to make correct judgments about the grammaticality of sentences. The self-paced reading results also revealed the L2 learners’ sensitivity to grammatical errors. Specifically, the Chinese Spanish L2ers were significantly slower in reading ungrammatical verbs than grammatical verbs, in the SV person agreement condition but not in the SV number agreement and adverb-verb TC conditions. The L2 speakers also read the three words following the verb significantly faster in the ungrammatical than in the grammatical sentences, regardless of the relation types. These results indicate that the L2 learners could detect feature mismatches between the verb and its related subject/adverb during self-paced reading. After detecting the verb mismatches (only slowdown at the verb in the SV person agreement condition), they achieved their goal and thus read the rest of the sentence at a faster rate to complete the grammaticality judgment task (for a similar interpretation see Leeser et al., 2011). Therefore, both the results of the self-paced reading and the grammaticality judgment task suggest that Chinese Spanish L2ers and native Spanish speakers show some similarities. But the Chinese Spanish L2ers in our study did not show a completely native-like knowledge of Spanish verbal inflection, despite their high accuracy rates.

The native Spanish speakers read the ungrammatical verbs significantly more slowly than the grammatical verbs in the SV number agreement condition, but not in the SV person agreement and the adverb-verb TC conditions. The lack of significant effects in the last two conditions may be a consequence of their intensive contact with Catalan in Barcelona. Catalan has two preterite paradigms (Comajoan, 2005), which are, respectively, labeled as simple preterite and periphrastic preterite (See Example 11). Both Catalan and Spanish use syncretic morphology to express their preterite tense (simple preterite). However, Catalan preterite is also expressed through a periphrastic construction based on an auxiliary (which has the verb *anar* ‘to go’ as its source) plus an infinitive.

11. Catalan Simple Preterite
  Ahir celebràrem_[1st Person] [PL] [PST]_ l’aniversari de l’Arnau.
  Yesterday celebrate the birthday of Arnau
  (‘Yesterday we celebrated Arnau’s birthday’)
  Catalan Periphrastic Preterite
  Ahir vam_[1st Person] [PL] [PST]_ celebrar l’aniversari de l’Arnau.
  Yesterday go-aux celebrate-inf the birthday of Arnau
  (‘Yesterday we celebrated Arnau’s birthday’)

Since most of our native Spanish subjects studied in a Catalan–Spanish bilingual context, they were likely to be exposed to both types of verbal forms during daily conversation. Moreover, there was a past-marking adverb preceding the verb in half of our experimental sentences. It is thus possible that both types of preterite forms could have been activated during the processing of the verb preceded by a past-marking adverb, thus leading to difficulties about the verb form to be expected and related difficulties during the processing adverb-verb TC and SV person agreement violations, but the basic grammatical mechanisms still underlay the processing. Our results are consistent with Enrique-Arias’s (2020) study showing that Catalan-Spanish bilinguals have difficulty during the production of Spanish standard preterite forms and are prone to extend the most frequently used suffix (3rd person suffix-o) to 1st person contexts. Nevertheless, the native Spanish speakers in the current study read the second or the third word following the verb significantly faster in the ungrammatical than in the grammatical sentences in the SV person agreement and adverb-verb TC conditions, suggesting that they can detect inflectional [Person] and [Tense] errors.

In general, our results suggest that both advanced Chinese Spanish L2ers and native Spanish speakers can put their grammatical knowledge of verbal inflection into use during real-time sentence reading, and make an accurate grammaticality judgment after reading a sentence.

### 7.2. Chinese Spanish L2ers process agreement and concord differently

The second research question investigates the processing mechanisms underlying agreement and concord. The reading time data of the Chinese Spanish L2ers showed that SV (person) agreement violations were detected earlier than adverb-verb TC violations. TC violations triggered speed-up effects at all the three words following the verb and SV (person) violations caused an additional slowdown effect at the target verb. This result may reflect that SV agreement and adverb-verb TC are different in nature (primary vs. non-primary and syntactic vs. discourse-related, as in Frazier and Clifton, 1996 and Biondo et al., 2018). Furthermore, there was a general tendency to judge adverb-verb TC relations less accurately than SV agreement relations, indicating a dissociation between these two relations for both the advanced Chinese Spanish L2ers and the native Spanish speakers. Compared to the previous study on English Spanish L2ers (Biondo and Mancini, 2021), the current study extends the relative influence of agreement and concord on L2 acquisition to a different bilingual population whose L1 lacks related morphemes.

Interestingly, the self-paced reading results of the native Spanish speakers showed that SV number (not person) agreement violations were detected earlier than adverb-verb TC violations in both the local and distal configurations. It is partially consistent with Biondo et al.’ (2018) eye-movement findings, in which adverb-verb TC violations were detected later than SV agreement violations only in the local configuration. The current results have not replicated the influence of linear distance on the processing of verbal inflection. However, this discrepancy can be attributed to methodological differences in the way the stimuli were presented. Whereas we presented each sentence in a word-by-word fashion, Biondo et al. (2018) displayed the entire sentence all at once and recorded eye movements. Since our readers are not allowed to read backward, they need to carefully comprehend and remember each word to process the relationship between the verb and the subject/adverb. The impossibility to reread the sentence may have made configuration effects less relevant. For this, an anonymous reviewer pointed out that the working memory should play a role in maintaining the information and we should consider the differences in cognitive costs caused by the (im) possibility to read backward. In this view, readers in the current study may experience a higher cognitive cost compared to those in Biondo et al.’ (2018) study, especially when dealing with the distal relations. And this relatively high cognitive cost may reduce the influence of the distal configuration in the current study.

Taken together, our data indicate that both advanced Chinese Spanish L2ers and native Spanish speakers can process SV agreement more efficiently than adverb-verb TC, though other factors may attenuate this difference.

### 7.3. Chinese Spanish L2ers process number agreement less efficiently than person agreement

The last question targets the specific role of different morphosyntactic features on L2 acquisition. The data of Chinese Spanish L2ers did not show online processing differences between SV number agreement and adverb-verb TC, although they did show a significant difference between SV person agreement and adverb-verb TC, as mentioned in the previous section. This finding may indicate that our learners had some difficulties with the processing of [Number] features, in line with previous studies of L2 acquisition (e.g., Jiang, 2004; Shibuya and Wakabayashi, 2008).

It is highly possible that the processing of L2 verbal inflection is shaped by L1-L2 similarity. The current study used a third person singular animate noun/determiner phrase (e.g., *el viajero cansado*, “the tired traveler”) as the subject when manipulating SV agreement. In Chinese, this kind of noun/determiner phrase can clearly express the value of the [Person] feature (i.e., [3rd Person]), but it can also be understood as a plural phrase even without the marker *mén* (e.g., 游客(们)来了 “tourists are coming”). Therefore, Chinese Spanish L2ers need to overcome negative transfer to compute SV number agreement, but show positive transfer to compute SV person agreement, leading the computation of number agreement to be less efficient than the computation of person agreement.

Another possibility is that the interpretive properties of the related features may play a role in L2 acquisition. The interpretation of person features requires a matching relation between the morphosyntactic person values (1st, 2nd, and 3rd person) and the speech participant values (Speaker, Addressee) encoded in the discourse representation of the sentence. Conversely, the interpretation of number features relies on a nominal/pronominal argument to express the cardinality of discourse referents (a single entity vs. a plurality; *cf.* Mancini et al., 2013). In other words, the anchoring points are different for [Number] and [Person] features, with the [Number] feature locating within the syntactic structure of the clause (i.e., the inflection layer) and the [Person] feature residing at the more peripheral discourse representation of the clause (i.e., the left periphery). This difference has been found to affect L1 processing consistently in self-paced reading (Mancini et al., 2014), eye-movements (Biondo et al., 2018), and ERP studies (Mancini et al., 2011; Zawiszewski et al., 2016). More importantly, Clahsen and Felser (2018) claim that late L2ers may assign more weight to discourse-based cues when compared to L1 speakers during sentence processing. In other words, the acquisition of the discourse-related features may thus be facilitated. Chinese Spanish L2ers may access/inspect the interpretive anchor more easily for [Person] than for [Number], leading to processing differences between person and number agreement.

In summary, both negative transfer from the L1 and the interpretative properties of number features may have affected the processing abilities of the advanced Chinese Spanish L2ers, leading to less efficient processing of SV number agreement compared to SV person agreement.

## 8. Conclusion

The present self-paced reading study demonstrates that both native Spanish speakers and Chinese Spanish L2ers can use different mechanisms to process SV agreement and adverb-verb TC. Agreement violations were detected earlier than concord violations. Thus, it is pertinent to emphasize morphosyntactic agreement as a primary linguistic computation and TC as a non-primary one, even though both computations facilitate language acquisition.

Moreover, Chinese Spanish L2ers appear to have difficulty with the processing of [Number] features. SV number agreement was processed less efficiently than SV person agreement, and possibly as efficient as adverb-verb TC. Yet, the grammaticality of SV agreement was generally judged more accurately than the grammaticality of adverb-verb TC. Some differences could thus still exist during the computation of [Number] and [Tense] features, as shown in previous L2 acquisition studies (Biondo and Mancini, 2021). Future studies should adopt other techniques such as eye tracking or ERPs to reveal these subtle differences.

## Data availability statement

The datasets presented in this study can be found in online repositories. The names of the repository/repositories and accession number(s) can be found in the article/Supplementary material.

## Ethics statement

The studies involving human participants were reviewed and approved by The Ethics Committee of Soochow University. The patients/participants provided their written informed consent to participate in this study.

## Author contributions

TM designed the research. ZZ performed experiments and analyzed data. TM, NB, and ZZ discussed and interpreted findings and wrote the paper. All authors contributed to the article and approved the submitted version.

## Funding

This research was supported by National Social Science Fund of China (21FYYB011) and Young Scholar Incubation Plan of Xizang Minzu University (20MDX01).

## Conflict of interest

The authors declare that the research was conducted in the absence of any commercial or financial relationships that could be construed as a potential conflict of interest.

## Publisher’s note

All claims expressed in this article are solely those of the authors and do not necessarily represent those of their affiliated organizations, or those of the publisher, the editors and the reviewers. Any product that may be evaluated in this article, or claim that may be made by its manufacturer, is not guaranteed or endorsed by the publisher.

## Footnotes

^1^The following abbreviations are used in this article: F = feminine; FUT = future tense; NH = nonhuman; PL = plural; PRS = present tense; PST = past tense; M = masculine; SG = singular.

^2^An anonymous reviewer questioned whether our study tested grammatical mechanisms or knowledge, or both. By holding an integrative view about the grammar/parser relationship, we believe that investigating the grammatical processing mechanisms employed by the L2ers is an appropriate way to check whether they have acquired the relevant grammatical knowledge, since the smoothness of the grammatical processing is dependent on the mastering of relevant grammatical knowledge. If L2ers have fully acquired the knowledge of SV agreement and adverb-verb TC relations, they can perform the syntactic operations (such as the Agree operation and other feature-based processing mechanisms) in a native-like fashion, so that the two constituents that have the matched feature are correctly related. Conversely, if the L2ers fail to perfom the operation, their performance should be not as tacit as native speakers’ performance. Both the grammatical knowledge and the parsing mechanisms are tested in the current study.

^3^As a quick survey shows, the native Spanish speakers were growing up with both Spanish and Catalan. And they have also learned English in classroom and other environments, reaching B1 level English. In this case, they are trilingual speakers.

^4^The Chinese Spanish L2ers did not major in English and did not use English in daily communication. However, they all passed the national College English Test Band 4 (CET-4), roughly equal to the International English Language Testing System (IELTS) scores of 4.5–5, a low-intermediate English proficiency level. Therefore, they were recognized as with comparatively low English proficiency and can also be viewed as trilingual speakers.

^5^One anonymous reviewer asked why we did not use the singular subject across conditions to make them comparable. On the one hand, the use of plural subjects in the SV agreement conditions would have been problematic because of “unagreement,” a linguistic phenomenon that allows 1st and 2nd person plural verbs to be grammatical in presence of 3rd person subjects in Spanish (e.g., Los viajeros regresamos/regresáis, “The travelers (we/you) come back”). On the other hand, the use of singular subjects in the adverb-tense TC conditions would have been problematic in terms of difference in verb length (e.g., sg, past: regresó; sg, future: regresará). Therefore, singular subjects were used for SV agreement manipulations while plural subjects were used for adverb-tense TC manipulations. Moreover, although the verb length was partially controlledduring the adverb-tense TC manipulations, there were still subtle differences across conditions, which stemmed from the original material used in the previous study. However, this should be not a problem for the statistical analyses (see Footnote 7 in Biondo, 2017, p. 135).

^6^As each presentation list had only 2–4 Chinese Spanish L2ers or native Spanish speakers to read the sentences, one may speculate that the performance of a single participant can change the data pattern of a list dramatically. To test this possibility, we ran statistical analyses including the list number as an additional fixed effect. The inclusion of interactions (e.g., list × grammaticality and/or list × language group) only influenced the self-paced reading results of regions *Verb +1* and *Verb +2*, but not that of *Verb* and *Verb +3*. Descriptive results of *Verb +1* and *Verb +2* showed that Chinese Spanish L2ers of some lists induced a larger speed-up effect (shorter RTs for the ungrammatical sentences compared with the grammatical sentences), compared with those of other lists. Since these results are not relevant to the research questions, they will not be discussed further.

^7^Because of the Covid-19 pandemic, we follow Lee and Shin (2016) to adopt the web platform Ibex Farm to examine native Spanish speakers remotely and the E-Prime software to test Chinese Spanish L2ers individually in a quiet room.
