# Supplementary material for: Adult Chinese Spanish L2ers’ acquisition of phi-agreement and temporal concord: The role of morphosyntactic features and adverb/subject-verb distance
Source: Front Psychol. 2022 Dec 22;13:1007828. doi: 10.3389/fpsyg.2022.1007828 (PMC9813440; doi:10.3389/fpsyg.2022.1007828)
Supplement: Supplementary file 1 [file Table_1.DOCX]

Table S1. The list of all experimental items.

| Item | Sentence |
| --- | --- |
| 1a | La próxima semana el viejo alcalde abandonará/*abandonarán/*abandonarás la oficina en el municipio. |
| 1b | Los viejos alcaldes la próxima semana abandonarán/*abandonaron la oficina en el municipio. |
| 1c | El viejo alcalde la próxima semana abandonará/*abandonarán/*abandonarás la oficina en el municipio. |
| 1d | La próxima semana los viejos alcaldes abandonarán/*abandonaron la oficina en el municipio. |
| 2a | El próximo año la vieja actriz abrirá/*abrirán/*abrirás una academia de teatro muy importante. |
| 2b | Las viejas actrices el próximo año abrirán/*abrieron una academia de teatro muy importante. |
| 2c | La vieja actriz el próximo año abrirá/*abrirán/*abrirás una academia de teatro muy importante. |
| 2d | El próximo año las viejas actrices abrirán/*abrieron una academia de teatro muy importante. |
| 3a | En dos meses la profesora acompañará/*acompañarán/*acompañarás a los estudiantes en una excursión. |
| 3b | Las profesoras en dos meses acompañarán/*acompañaron a los estudiantes en una excursión. |
| 3c | La profesora en dos meses acompañará/*acompañarán/*acompañarás a los estudiantes en una excursión. |
| 3d | En dos meses las profesoras acompañarán/*acompañaron a los estudiantes en una excursión. |
| 4a | La próxima semana la vecina de casa adoptará/*adoptarán/*adoptarás a un nuevo gatito. |
| 4b | Las vecinas de casa la próxima semana adoptarán/*adoptaron a un nuevo gatito. |
| 4c | La vecina de casa la próxima semana adoptará/*adoptarán/*adoptarás a un nuevo gatito. |
| 4d | La próxima semana las vecinas de casa adoptarán/*adoptaron a un nuevo gatito. |
| 5a | Mañana temprano el comandante alcanzará/*alcanzarán/*alcanzarás la nueva base militar. |
| 5b | Los comandantes mañana temprano alcanzarán/*alcanzaron la nueva base militar. |
| 5c | El comandante mañana temprano alcanzará/*alcanzarán/*alcanzarás la nueva base militar. |
| 5d | Mañana temprano los comandantes alcanzarán/*alcanzaron la nueva base militar. |
| 6a | Mañana a las dos el crítico de cine almorzará/*almorzarán/*almorzarás con un famoso director americano. |
| 6b | Los críticos de cine mañana a las dos almorzarán/*almorzaron con un famoso director americano. |
| 6c | El crítico de cine mañana a las dos almorzará/*almorzarán/*almorzarás con un famoso director americano. |
| 6d | Mañana a las dos los críticos de cine almorzarán/*almorzaron con un famoso director americano. |
| 7a | El próximo invierno el masajista del balneario aplicará/*aplicarán/*aplicarás un nuevo masaje. |
| 7b | Los masajistas del balneario el próximo invierno aplicarán/*aplicaron un nuevo masaje. |
| 7c | El masajista del balneario el próximo invierno aplicará/*aplicarán/*aplicarás un nuevo masaje. |
| 7d | El próximo invierno los masajistas del balneario aplicarán/*aplicaron un nuevo masaje. |
| 8a | Mañana al mediodía el amigo soltero asistirá/*asistirán/*asistirás a la boda de dos amigos ricos. |
| 8b | Los amigos solteros mañana al mediodía asistirán/*asistieron a la boda de dos amigos ricos. |
| 8c | El amigo soltero mañana al mediodía asistirá/*asistirán/*asistirás a la boda de dos amigos ricos. |
| 8d | Mañana al mediodía los amigos solteros asistirán/*asistieron a la boda de dos amigos ricos. |
| 9a | En dos días el marinero atacará/*atacarán/*atacarás el buque mercante. |
| 9b | Los marineros en dos días atacarán/*atacaron el buque mercante. |
| 9c | El marinero en dos días atacará/*atacarán/*atacarás el buque mercante. |
| 9d | En dos días los marineros atacarán/*atacaron el buque mercante. |
| 10a | El próximo año el sacerdote casará/*casarán/*casarás a los novios en la catedral de Sevilla. |
| 10b | Los sacerdotes el próximo año casarán/*casaron a los novios en la catedral de Sevilla. |
| 10c | El sacerdote el próximo año casará/*casarán/*casarás a los novios en la catedral de Sevilla. |
| 10d | El próximo año los sacerdotes casarán/*casaron a los novios en la catedral de Sevilla. |
| 11a | La próxima semana el dueño del pub cerrará/*cerrarán/*cerrarás la barra más tarde. |
| 11b | Los dueños del pub la próxima semana cerrarán/*cerraron la barra más tarde. |
| 11c | El dueño del pub la próxima semana cerrará/*cerrarán/*cerrarás la barra más tarde. |
| 11d | La próxima semana los dueños del pub cerrarán/*cerraron la barra más tarde. |
| 12a | El próximo mes el millonario comerá/*comerán/*comerás en el nuevo restaurante de lujo en Dubái. |
| 12b | Los millonarios el próximo mes comerán/*comieron en el nuevo restaurante de lujo en Dubái. |
| 12c | El millonario el próximo mes comerá/*comerán/*comerás en el nuevo restaurante de lujo en Dubái. |
| 12d | El próximo mes los millonarios comerán/*comieron en el nuevo restaurante de lujo en Dubái. |
| 13a | Mañana al mediodía el juez de la corte comunicará/*comunicarán/*comunicarás la sentencia al demandado. |
| 13b | Los jueces de la corte mañana al mediodía comunicarán/*comunicaron la sentencia al demandado. |
| 13c | El juez de la corte mañana al mediodía comunicará/*comunicarán/*comunicarás la sentencia al demandado. |
| 13d | Mañana al mediodía los jueces de la corte comunicarán/*comunicaron la sentencia al demandado. |
| 14a | Mañana al mediodía el autor del libro concederá/*concederán/*concederás una larga entrevista. |
| 14b | Los autores del libro mañana al mediodía concederán/*concedieron una larga entrevista. |
| 14c | El autor del libro mañana al mediodía concederá/*concederán/*concederás una larga entrevista. |
| 14d | Mañana al mediodía los autores del libro concederán/*concedieron una larga entrevista. |
| 15a | Mañana al mediodía el empleado público conocerá/*conocerán/*conocerás al nuevo director general. |
| 15b | Los empleados públicos mañana al mediodía conocerán/*conocieron al nuevo director general. |
| 15c | El empleado público mañana al mediodía conocerá/*conocerán/*conocerás al nuevo director general. |
| 15d | Mañana al mediodía los empleados públicos conocerán/*conocieron al nuevo director general. |
| 16a | Mañana por la noche el veterano de guerra contará/*contarán/*contarás sus experiencias en Vietnam. |
| 16b | Los veteranos de guerra mañana por la noche contarán/*contaron sus experiencias en Vietnam. |
| 16c | El veterano de guerra mañana por la noche contará/*contarán/*contarás sus experiencias en Vietnam. |
| 16d | Mañana por la noche los veteranos de guerra contarán/*contaron sus experiencias en Vietnam. |
| 17a | El próximo año el corredor correrá/*correrán/*correrás la famosa maratón de New York. |
| 17b | Los corredores el próximo año correrán/*corrieron la famosa maratón de New York. |
| 17c | El corredor el próximo año correrá/*correrán/*correrás la famosa maratón de New York. |
| 17d | El próximo año los corredores correrán/*corrieron la famosa maratón de New York. |
| 18a | En algunos días el universitario dirigirá/*dirigirán/*dirigirás la manifestación contra el ministerio. |
| 18b | Los universitarios en algunos días dirigirán/*dirigieron la manifestación contra el ministerio. |
| 18c | El universitario en algunos días dirigirá/*dirigirán/*dirigirás la manifestación contra el ministerio. |
| 18d | En algunos días los universitarios dirigirán/*dirigieron la manifestación contra el ministerio. |
| 19a | Mañana por la tarde el vendedor de telas dispondrá/*dispondrán/*dispondrás la mercancía en las casetas. |
| 19b | Los vendedores de telas mañana por la tarde dispondrán/*dispusieron la mercancía en las casetas. |
| 19c | El vendedor de telas mañana por la tarde dispondrá/*dispondrán/*dispondrás la mercancía en las casetas. |
| 19d | Mañana por la tarde los vendedores de telas dispondrán/*dispusieron la mercancía en las casetas. |
| 20a | El próximo mes el escenógrafo encontrará/*encontrarán/*encontrarás a los técnicos de iluminación. |
| 20b | Los escenógrafos el próximo mes encontrarán/*encontraron a los técnicos de iluminación. |
| 20c | El escenógrafo el próximo mes encontrará/*encontrarán/*encontrarás a los técnicos de iluminación. |
| 20d | El próximo mes los escenógrafos encontrarán/*encontraron a los técnicos de iluminación. |
| 21a | La próxima semana el nuevo cartero entregará/*entregarán/*entregarás el correo sólo en el centro. |
| 21b | Los nuevos carteros la próxima semana entregarán/*entregaron el correo sólo en el centro. |
| 21c | El nuevo cartero la próxima semana entregará/*entregarán/*entregarás el correo sólo en el centro. |
| 21d | La próxima semana los nuevos carteros entregarán/*entregaron el correo sólo en el centro. |
| 22a | La próxima semana el psicoterapeuta enviará/*enviarán/*enviarás una carta a la comisión. |
| 22b | Los psicoterapeutas la próxima semana enviarán/*enviaron una carta a la comisión. |
| 22c | El psicoterapeuta la próxima semana enviará/*enviarán/*enviarás una carta a la comisión. |
| 22d | La próxima semana los psicoterapeutas enviarán/*enviaron una carta a la comisión. |
| 23a | En algunos días el soldado examinará/*examinarán/*examinarás el mapa de las zonas más peligrosas. |
| 23b | Los soldados en algunos días examinarán/*examinaron el mapa de las zonas más peligrosas. |
| 23c | El soldado en algunos días examinará/*examinarán/*examinarás el mapa de las zonas más peligrosas. |
| 23d | En algunos días los soldados examinarán/*examinaron el mapa de las zonas más peligrosas. |
| 24a | El próximo mes el presidente firmará/*firmarán/*firmarás un tratado de paz. |
| 24b | Los presidentes el próximo mes firmarán/*firmaron un tratado de paz. |
| 24c | El presidente el próximo mes firmará/*firmarán/*firmarás un tratado de paz. |
| 24d | El próximo mes los presidentes firmarán/*firmaron un tratado de paz. |
| 25a | En seis meses el astronauta grabará/*grabarán/*grabarás nuevas imágenes de Marte. |
| 25b | Los astronautas en seis meses grabarán/*grabaron nuevas imágenes de Marte. |
| 25c | El astronauta en seis meses grabará/*grabarán/*grabarás nuevas imágenes de Marte. |
| 25d | En seis meses los astronautas grabarán/*grabaron nuevas imágenes de Marte. |
| 26a | En seis meses el lingüista hablará/*hablarán/*hablarás en una conferencia en Potsdam. |
| 26b | Los lingüistas en seis meses hablarán/*hablaron en una conferencia en Potsdam. |
| 26c | El lingüista en seis meses hablará/*hablarán/*hablarás en una conferencia en Potsdam. |
| 26d | En seis meses los lingüistas hablarán/*hablaron en una conferencia en Potsdam. |
| 27a | Mañana por la tarde el colega de trabajo informará/*informarán/*informarás al empleado del nuevo contrato. |
| 27b | Los colegas de trabajo mañana por la tarde informarán/*informaron al empleado del nuevo contrato. |
| 27c | El colega de trabajo mañana por la tarde informará/*informarán/*informarás al empleado del nuevo contrato. |
| 27d | Mañana por la tarde los colegas de trabajo informarán/*informaron al empleado del nuevo contrato. |
| 28a | Mañana durante la sesión el psicoanalista interpretará/*interpretarán/*interpretarás el sueño del cliente. |
| 28b | Los psicoanalistas mañana durante la sesión interpretarán/*interpretaron el sueño del cliente. |
| 28c | El psicoanalista mañana durante la sesión interpretará/*interpretarán/*interpretarás el sueño del cliente. |
| 28d | Mañana durante la sesión los psicoanalistas interpretarán/*interpretaron el sueño del cliente. |
| 29a | Mañana a las dos el niño deportista jugará/*jugarán/*jugarás al fútbol con sus amigos. |
| 29b | Los niños deportistas mañana a las dos jugarán/*jugaron al fútbol con sus amigos. |
| 29c | El niño deportista mañana a las dos jugará/*jugarán/*jugarás al fútbol con sus amigos. |
| 29d | Mañana a las dos los niños deportistas jugarán/*jugaron al fútbol con sus amigos. |
| 30a | Mañana temprano el limpiador lavará/*lavarán/*lavarás el suelo de la sala de actos. |
| 30b | Los limpiadores mañana temprano lavarán/*lavaron el suelo de la sala de actos. |
| 30c | El limpiador mañana temprano lavará/*lavarán/*lavarás el suelo de la sala de actos. |
| 30d | Mañana temprano los limpiadores lavarán/*lavaron el suelo de la sala de actos. |
| 31a | En dos días el notario leerá/*leerán/*leerás el testamento delante de todos los herederos. |
| 31b | Los notarios en dos días leerán/*leyeron el testamento delante de todos los herederos. |
| 31c | El notario en dos días leerá/*leerán/*leerás el testamento delante de todos los herederos. |
| 31d | En dos días los notarios leerán/*leyeron el testamento delante de todos los herederos. |
| 32a | Mañana el invitado llevará/*llevarán/*llevarás una botella de vino tinto. |
| 32b | Los invitados mañana llevarán/*llevaron una botella de vino tinto. |
| 32c | El invitado mañana llevará/*llevarán/*llevarás una botella de vino tinto. |
| 32d | Mañana los invitados llevarán/*llevaron una botella de vino tinto. |
| 33a | Mañana por la noche el piloto español luchará/*lucharán/*lucharás por la victoria del Gran Premio. |
| 33b | Los pilotos españoles mañana por la noche lucharán/*lucharon por la victoria del Gran Premio. |
| 33c | El piloto español mañana por la noche luchará/*lucharán/*lucharás por la victoria del Gran Premio. |
| 33d | Mañana por la noche los pilotos españoles lucharán/*lucharon por la victoria del Gran Premio. |
| 34a | El próximo mes el graduado mandará/*mandarán/*mandarás la versión final de la tesis. |
| 34b | Los graduados el próximo mes mandarán/*mandaron la versión final de la tesis. |
| 34c | El graduado el próximo mes mandará/*mandarán/*mandarás la versión final de la tesis. |
| 34d | El próximo mes los graduados mandarán/*mandaron la versión final de la tesis. |
| 35a | La próxima semana el embajador ruso negociará/*negociarán/*negociarás un nuevo acuerdo comercial. |
| 35b | Los embajadores rusos la próxima semana negociarán/*negociaron un nuevo acuerdo comercial. |
| 35c | El embajador ruso la próxima semana negociará/*negociarán/*negociarás un nuevo acuerdo comercial. |
| 35d | La próxima semana los embajadores rusos negociarán/*negociaron un nuevo acuerdo comercial. |
| 36a | Mañana por la noche el teniente coronel ocupará/*ocuparán/*ocuparás la base enemiga más peligrosa. |
| 36b | Los tenientes coronel mañana por la noche ocuparán/*ocuparon la base enemiga más peligrosa. |
| 36c | El teniente coronel mañana por la noche ocupará/*ocuparán/*ocuparás la base enemiga más peligrosa. |
| 36d | Mañana por la noche los tenientes coronel ocuparán/*ocuparon la base enemiga más peligrosa. |
| 37a | Mañana por la tarde la nueva secretaria ordenará/*ordenarán/*ordenarás los archivos del bufete. |
| 37b | Las nuevas secretarias mañana por la tarde ordenarán/*ordenaron los archivos del bufete. |
| 37c | La nueva secretaria mañana por la tarde ordenará/*ordenarán/*ordenarás los archivos del bufete. |
| 37d | Mañana por la tarde las nuevas secretarias ordenarán/*ordenaron los archivos del bufete. |
| 38a | En dos meses el jubilado participará/*participarán/*participarás en un torneo de bolos. |
| 38b | Los jubilados en dos meses participarán/*participaron en un torneo de bolos. |
| 38c | El jubilado en dos meses participará/*participarán/*participarás en un torneo de bolos. |
| 38d | En dos meses los jubilados participarán/*participaron en un torneo de bolos. |
| 39a | Mañana el vegetariano podrá/*podrán/*podrás escoger entre varios platos sin carne. |
| 39b | Los vegetarianos mañana podrán/*pudieron escoger entre varios platos sin carne. |
| 39c | El vegetariano mañana podrá/*podrán/*podrás escoger entre varios platos sin carne. |
| 39d | Mañana los vegetarianos podrán/*pudieron escoger entre varios platos sin carne. |
| 40a | Mañana por la tarde la bailarina de tango probará/*probarán/*probarás la nueva coreografía. |
| 40b | Las bailarinas de tango mañana por la tarde probarán/*probaron la nueva coreografía. |
| 40c | La bailarina de tango mañana por la tarde probará/*probarán/*probarás la nueva coreografía. |
| 40d | Mañana por la tarde las bailarinas de tango probarán/*probaron la nueva coreografía. |
| 41a | Mañana al mediodía el viejo trabajador protestará/*protestarán/*protestarás enfrente de la empresa. |
| 41b | Los viejos trabajadores mañana al mediodía protestarán/*protestaron enfrente de la empresa. |
| 41c | El viejo trabajador mañana al mediodía protestará/*protestarán/*protestarás enfrente de la empresa. |
| 41d | Mañana al mediodía los viejos trabajadores protestarán/*protestaron enfrente de la empresa. |
| 42a | Mañana temprano el vigilante quitará/*quitarán/*quitarás los carteles del muro. |
| 42b | Los vigilantes mañana temprano quitarán/*quitaron los carteles del muro. |
| 42c | El vigilante mañana temprano quitará/*quitarán/*quitarás los carteles del muro. |
| 42d | Mañana temprano los vigilantes quitarán/*quitaron los carteles del muro. |
| 43a | Mañana temprano el barrendero recogerá/*recogerán/*recogerás la basura sólo en el centro. |
| 43b | Los barrenderos mañana temprano recogerán/*recogieron la basura sólo en el centro. |
| 43c | El barrendero mañana temprano recogerá/*recogerán/*recogerás la basura sólo en el centro. |
| 43d | Mañana temprano los barrenderos recogerán/*recogieron la basura sólo en el centro. |
| 44a | Mañana el camionero recorrerá/*recorrerán/*recorrerás más de doscientos kilómetros. |
| 44b | Los camioneros mañana recorrerán/*recorrieron más de doscientos kilómetros. |
| 44c | El camionero mañana recorrerá/*recorrerán/*recorrerás más de doscientos kilómetros. |
| 44d | Mañana los camioneros recorrerán/*recorrieron más de doscientos kilómetros. |
| 45a | La próxima semana el nuevo dirigente reemplazará/*reemplazarán/*reemplazarás los empleados vagos. |
| 45b | Los nuevos dirigentes la próxima semana reemplazarán/*reemplazaron los empleados vagos. |
| 45c | El nuevo dirigente la próxima semana reemplazará/*reemplazarán/*reemplazarás los empleados vagos. |
| 45d | La próxima semana los nuevos dirigentes reemplazarán/*reemplazaron los empleados vagos. |
| 46a | En seis meses el explorador regresará/*regresarán/*regresarás de un largo viaje en África. |
| 46b | Los exploradores en seis meses regresarán/*regresaron de un largo viaje en África. |
| 46c | El explorador en seis meses regresará/*regresarán/*regresarás de un largo viaje en África. |
| 46d | En seis meses los exploradores regresarán/*regresaron de un largo viaje en África. |
| 47a | El próximo año el arquitecto revisará/*revisarán/*revisarás una parte del proyecto. |
| 47b | Los arquitectos el próximo año revisarán/*revisaron una parte del proyecto. |
| 47c | El arquitecto el próximo año revisará/*revisarán/*revisarás una parte del proyecto. |
| 47d | El próximo año los arquitectos revisarán/*revisaron una parte del proyecto. |
| 48a | La próxima semana el pintor famoso traerá/*traerán/*traerás los cuadros a la galería. |
| 48b | Los pintores famosos la próxima semana traerán/*trajeron los cuadros a la galería. |
| 48c | El pintor famoso la próxima semana traerá/*traerán/*traerás los cuadros a la galería. |
| 48d | La próxima semana los pintores famosos traerán/*trajeron los cuadros a la galería. |
| 49a | Mañana temprano el agricultor venderá/*venderán/*venderás los productos en el mercado. |
| 49b | Los agricultores mañana temprano venderán/*vendieron los productos en el mercado. |
| 49c | El agricultor mañana temprano venderá/*venderán/*venderás los productos en el mercado. |
| 49d | Mañana temprano los agricultores venderán/*vendieron los productos en el mercado. |
| 50a | Mañana por la noche el agente de policía vigilará/*vigilarán/*vigilarás la periferia de la ciudad. |
| 50b | Los agentes de policía mañana por la noche vigilarán/*vigilaron la periferia de la ciudad. |
| 50c | El agente de policía mañana por la noche vigilará/*vigilarán/*vigilarás la periferia de la ciudad. |
| 50d | Mañana por la noche los agentes de policía vigilarán/*vigilaron la periferia de la ciudad. |
| 51a | Ayer al mediodía la modista joven acudió/*acudieron/*acudiste a la clase de bordado. |
| 51b | Las modistas jóvenes ayer al mediodía acudieron/*acudirán a la clase de bordado. |
| 51c | La modista joven ayer al mediodía acudió/*acudieron/*acudiste a la clase de bordado. |
| 51d | Ayer al mediodía las modistas jóvenes acudieron/*acudirán a la clase de bordado. |
| 52a | Hace algunos días la hábil pastelera adornó/*adornaron/*adornaste las tartas de chocolate. |
| 52b | Las hábiles pasteleras hace algunos días adornaron/*adornarán las tartas de chocolate. |
| 52c | La hábil pastelera hace algunos días adornó/*adornaron/*adornaste las tartas de chocolate. |
| 52d | Hace algunos días las hábiles pasteleras adornaron/*adornarán las tartas de chocolate. |
| 53a | Anteayer el senador aprobó/*aprobaron/*aprobaste la nueva ley. |
| 53b | Los senadores anteayer aprobaron/*aprobarán la nueva ley. |
| 53c | El senador anteayer aprobó/*aprobaron/*aprobaste la nueva ley. |
| 53d | Anteayer los senadores aprobaron/*aprobarán la nueva ley. |
| 54a | El mes pasado el jardinero arrancó/*arrancaron/*arrancaste las malas hierbas del jardín. |
| 54b | Los jardineros el mes pasado arrancaron/*arrancarán las malas hierbas del jardín. |
| 54c | El jardinero el mes pasado arrancó/*arrancaron/*arrancaste las malas hierbas del jardín. |
| 54d | El mes pasado los jardineros arrancaron/*arrancarán las malas hierbas del jardín. |
| 55a | Ayer por la tarde el poeta sudamericano atrajo/*atrajeron/*atrajiste la atención de los críticos. |
| 55b | Los poetas sudamericanos ayer por la tarde atrajeron/*atraerán la atención de los críticos. |
| 55c | El poeta sudamericano ayer por la tarde atrajo/*atrajeron/*atrajiste la atención de los críticos. |
| 55d | Ayer por la tarde los poetas sudamericanos atrajeron/*atraerán la atención de los críticos. |
| 56a | Hace un mes el banquero aumentó/*aumentaron/*aumentaste el tipo de interés anual. |
| 56b | Los banqueros hace un mes aumentaron/*aumentarán el tipo de interés anual. |
| 56c | El banquero hace un mes aumentó/*aumentaron/*aumentaste el tipo de interés anual. |
| 56d | Hace un mes los banqueros aumentaron/*aumentarán el tipo de interés anual. |
| 57a | Hace un mes el neurólogo buscó/*buscaron/*buscaste un nuevo empleo en una clínica privada. |
| 57b | Los neurólogos hace un mes buscaron/*buscarán un nuevo empleo en una clínica privada. |
| 57c | El neurólogo hace un mes buscó/*buscaron/*buscaste un nuevo empleo en una clínica privada. |
| 57d | Hace un mes los neurólogos buscaron/*buscarán un nuevo empleo en una clínica privada. |
| 58a | Hace algunos días el enólogo experto cambió/*cambiaron/*cambiaste la carta de vinos. |
| 58b | Los enólogos expertos hace algunos días cambiaron/*cambiarán la carta de vinos. |
| 58c | El enólogo experto hace algunos días cambió/*cambiaron/*cambiaste la carta de vinos. |
| 58d | Hace algunos días los enólogos expertos cambiaron/*cambiarán la carta de vinos. |
| 59a | Hace un mes el escultor colocó/*colocaron/*colocaste las estatuas en el nuevo museo. |
| 59b | Los escultores hace un mes colocaron/*colocarán las estatuas en el nuevo museo. |
| 59c | El escultor hace un mes colocó/*colocaron/*colocaste las estatuas en el nuevo museo. |
| 59d | Hace un mes los escultores colocaron/*colocarán las estatuas en el nuevo museo. |
| 60a | Anteayer el detective completó/*completaron/*completaste el reconocimiento en el lugar del crimen. |
| 60b | Los detectives anteayer completaron/*completarán el reconocimiento en el lugar del crimen. |
| 60c | El detective anteayer completó/*completaron/*completaste el reconocimiento en el lugar del crimen. |
| 60d | Anteayer los detectives completaron/*completarán el reconocimiento en el lugar del crimen. |
| 61a | Anoche la chica compró/*compraron/*compraste unas prendas muy cara. |
| 61b | Las chicas anoche compraron/*comprarán unas prendas muy cara. |
| 61c | La chica anoche compró/*compraron/*compraste unas prendas muy cara. |
| 61d | Anoche las chicas compraron/*comprarán unas prendas muy cara. |
| 62a | Hace un mes el aprendiz consiguió/*consiguieron/*conseguiste un diploma de estudios avanzados. |
| 62b | Los aprendices hace un mes consiguieron/*conseguirán un diploma de estudios avanzados. |
| 62c | El aprendiz hace un mes consiguió/*consiguieron/*conseguiste un diploma de estudios avanzados. |
| 62d | Hace un mes los aprendices consiguieron/*conseguirán un diploma de estudios avanzados. |
| 63a | El verano pasado el misionero joven construyó/*construyeron/*construiste un pozo en Somalia. |
| 63b | Los misioneros jóvenes el verano pasado construyeron/*construirán un pozo en Somalia. |
| 63c | El misionero joven el verano pasado construyó/*construyeron/*construiste un pozo en Somalia. |
| 63d | El verano pasado los misioneros jóvenes construyeron/*construirán un pozo en Somalia. |
| 64a | Anoche el cómico contó/*contaron/*contaste sólo chistes de políticos italianos. |
| 64b | Los cómicos anoche contaron/*contarán sólo chistes de políticos italianos. |
| 64c | El cómico anoche contó/*contaron/*contaste sólo chistes de políticos italianos. |
| 64d | Anoche los cómicos contaron/*contarán sólo chistes de políticos italianos. |
| 65a | Anoche la maestra corrigió/*corrigieron/*corregiste las tareas de los alumnos. |
| 65b | Las maestras anoche corrigieron/*corregirán las tareas de los alumnos. |
| 65c | La maestra anoche corrigió/*corrigieron/*corregiste las tareas de los alumnos. |
| 65d | Anoche las maestras corrigieron/*corregirán las tareas de los alumnos. |
| 66a | El otoño pasado el leñador cortó/*cortaron/*cortaste mucha más madera. |
| 66b | Los leñadores el otoño pasado cortaron/*cortarán mucha más madera. |
| 66c | El leñador el otoño pasado cortó/*cortaron/*cortaste mucha más madera. |
| 66d | El otoño pasado los leñadores cortaron/*cortarán mucha más madera. |
| 67a | Hace algunos días el obrero despedido denunció/*denunciaron/*denunciaste al jefe de la compañía. |
| 67b | Los obreros despedidos hace algunos días denunciaron/*denunciarán al jefe de la compañía. |
| 67c | El obrero despedido hace algunos días denunció/*denunciaron/*denunciaste al jefe de la compañía. |
| 67d | Hace algunos días los obreros despedidos denunciaron/*denunciarán al jefe de la compañía. |
| 68a | El año pasado el empresario despidió/*despidieron/*despediste a todos los obreros de la empresa. |
| 68b | Los empresarios el año pasado despidieron/*despedirán a todos los obreros de la empresa. |
| 68c | El empresario el año pasado despidió/*despidieron/*despediste a todos los obreros de la empresa. |
| 68d | El año pasado los empresarios despidieron/*despedirán a todos los obreros de la empresa. |
| 69a | El mes pasado la feminista discutió/*discutieron/*discutiste sobre la violencia machista. |
| 69b | Las feministas el mes pasado discutieron/*discutirán sobre la violencia machista. |
| 69c | La feminista el mes pasado discutió/*discutieron/*discutiste sobre la violencia machista. |
| 69d | El mes pasado las feministas discutieron/*discutirán sobre la violencia machista. |
| 70a | Ayer temprano el viejo pescador echó/*echaron/*echaste las redes mar adentro. |
| 70b | Los viejos pescadores ayer temprano echaron/*echarán las redes mar adentro. |
| 70c | El viejo pescador ayer temprano echó/*echaron/*echaste las redes mar adentro. |
| 70d | Ayer temprano los viejos pescadores echaron/*echarán las redes mar adentro. |
| 71a | Anoche el jurado eligió/*eligieron/*elegiste la vencedora del concurso de belleza. |
| 71b | Los jurados anoche eligieron/*elegirán la vencedora del concurso de belleza. |
| 71c | El jurado anoche eligió/*eligieron/*elegiste la vencedora del concurso de belleza. |
| 71d | Anoche los jurados eligieron/*elegirán la vencedora del concurso de belleza. |
| 72a | El verano pasado el monitor de natación enseñó/*enseñaron/*enseñaste a nadar a los niños. |
| 72b | Los monitores de natación el verano pasado enseñaron/*enseñarán a nadar a los niños. |
| 72c | El monitor de natación el verano pasado enseñó/*enseñaron/*enseñaste a nadar a los niños. |
| 72d | El verano pasado los monitores de natación enseñaron/*enseñarán a nadar a los niños. |
| 73a | El mes pasado el diputado escogió/*escogieron/*escogiste un nuevo presidente. |
| 73b | Los diputados el mes pasado escogieron/*escogerán un nuevo presidente. |
| 73c | El diputado el mes pasado escogió/*escogieron/*escogiste un nuevo presidente. |
| 73d | El mes pasado los diputados escogieron/*escogerán un nuevo presidente. |
| 74a | La primavera pasada el librero viejo formó/*formaron/*formaste una asociación promotora de lectura. |
| 74b | Los libreros viejos la primavera pasada formaron/*formarán una asociación promotora de lectura. |
| 74c | El librero viejo la primavera pasada formó/*formaron/*formaste una asociación promotora de lectura. |
| 74d | La primavera pasada los libreros viejos formaron/*formarán una asociación promotora de lectura. |
| 75a | La semana pasada el concursante ganó/*ganaron/*ganaste el primer premio de la competición. |
| 75b | Los concursantes la semana pasada ganaron/*ganarán el primer premio de la competición. |
| 75c | El concursante la semana pasada ganó/*ganaron/*ganaste el primer premio de la competición. |
| 75d | La semana pasada los concursantes ganaron/*ganarán el primer premio de la competición. |
| 76a | El invierno pasado el pastor de ovejas hizo/*hicieron/*hiciste queso de forma artesanal. |
| 76b | Los pastores de ovejas el invierno pasado hicieron/*harán queso de forma artesanal. |
| 76c | El pastor de ovejas el invierno pasado hizo/*hicieron/*hiciste queso de forma artesanal. |
| 76d | El invierno pasado los pastores de ovejas hicieron/*harán queso de forma artesanal. |
| 77a | Hace un mes el ciclista inició/*iniciaron/*iniciaste el entrenamiento de fortalecimiento. |
| 77b | Los ciclistas hace un mes iniciaron/*iniciarán el entrenamiento de fortalecimiento. |
| 77c | El ciclista hace un mes inició/*iniciaron/*iniciaste el entrenamiento de fortalecimiento. |
| 77d | Hace un mes los ciclistas iniciaron/*iniciarán el entrenamiento de fortalecimiento. |
| 78a | El mes pasado el encarcelado intentó/*intentaron/*intentaste la fuga de la cárcel. |
| 78b | Los encarcelados el mes pasado intentaron/*intentarán la fuga de la cárcel. |
| 78c | El encarcelado el mes pasado intentó/*intentaron/*intentaste la fuga de la cárcel. |
| 78d | El mes pasado los encarcelados intentaron/*intentarán la fuga de la cárcel. |
| 79a | Ayer el cónsul invitó/*invitaron/*invitaste algunos empresarios a la embajada española. |
| 79b | Los cónsules ayer invitaron/*invitarán algunos empresarios a la embajada española. |
| 79c | El cónsul ayer invitó/*invitaron/*invitaste algunos empresarios a la embajada española. |
| 79d | Ayer los cónsules invitaron/*invitarán algunos empresarios a la embajada española. |
| 80a | Hace dos meses el presentador lideró/*lideraron/*lideraste un nuevo programa televisivo. |
| 80b | Los presentadores hace dos meses lideraron/*liderarán un nuevo programa televisivo. |
| 80c | El presentador hace dos meses lideró/*lideraron/*lideraste un nuevo programa televisivo. |
| 80d | Hace dos meses los presentadores lideraron/*liderarán un nuevo programa televisivo. |
| 81a | Ayer en la entrevista el jugador de baloncesto negó/*negaron/*negaste el uso de dopaje. |
| 81b | Los jugadores de baloncesto ayer en la entrevista negaron/*negarán el uso de dopaje. |
| 81c | El jugador de baloncesto ayer en la entrevista negó/*negaron/*negaste el uso de dopaje. |
| 81d | Ayer en la entrevista los jugadores de baloncesto negaron/*negarán el uso de dopaje. |
| 82a | Anteayer el obispo nombró/*nombraron/*nombraste al nuevo cardenal. |
| 82b | Los obispos anteayer nombraron/*nombrarán al nuevo cardenal. |
| 82c | El obispo anteayer nombró/*nombraron/*nombraste al nuevo cardenal. |
| 82d | Anteayer los obispos nombraron/*nombrarán al nuevo cardenal. |
| 83a | Anteayer el monje ofreció/*ofrecieron/*ofreciste alimentos a los pobres del barrio. |
| 83b | Los monjes anteayer ofrecieron/*ofrecerán alimentos a los pobres del barrio. |
| 83c | El monje anteayer ofreció/*ofrecieron/*ofreciste alimentos a los pobres del barrio. |
| 83d | Anteayer los monjes ofrecieron/*ofrecerán alimentos a los pobres del barrio. |
| 84a | Hace algunos meses el minero irlandés pidió/*pidieron/*pediste un sistema de recogida de carbón. |
| 84b | Los mineros irlandeses hace algunos meses pidieron/*pedirán un sistema de recogida de carbón. |
| 84c | El minero irlandés hace algunos meses pidió/*pidieron/*pediste un sistema de recogida de carbón. |
| 84d | Hace algunos meses los mineros irlandeses pidieron/*pedirán un sistema de recogida de carbón. |
| 85a | La semana pasada el campesino plantó/*plantaron/*plantaste berenjenas y pimientos. |
| 85b | Los campesinos la semana pasada plantaron/*plantarán berenjenas y pimientos. |
| 85c | El campesino la semana pasada plantó/*plantaron/*plantaste berenjenas y pimientos. |
| 85d | La semana pasada los campesinos plantaron/*plantarán berenjenas y pimientos. |
| 86a | El otoño pasado la monja joven prestó/*prestaron/*prestaste ayuda a las personas sin hogar. |
| 86b | Las monjas jóvenes el otoño pasado prestaron/*prestarán ayuda a las personas sin hogar. |
| 86c | La monja joven el otoño pasado prestó/*prestaron/*prestaste ayuda a las personas sin hogar. |
| 86d | El otoño pasado las monjas jóvenes prestaron/*prestarán ayuda a las personas sin hogar. |
| 87a | Anteayer el futbolista reclamó/*reclamaron/*reclamaste el gol anulado por el árbitro. |
| 87b | Los futbolistas anteayer reclamaron/*reclamarán el gol anulado por el árbitro. |
| 87c | El futbolista anteayer reclamó/*reclamaron/*reclamaste el gol anulado por el árbitro. |
| 87d | Anteayer los futbolistas reclamaron/*reclamarán el gol anulado por el árbitro. |
| 88a | Anteayer el abuelo regaló/*regalaron/*regalaste un coche muy caro al nieto. |
| 88b | Los abuelos anteayer regalaron/*regalarán un coche muy caro al nieto. |
| 88c | El abuelo anteayer regaló/*regalaron/*regalaste un coche muy caro al nieto. |
| 88d | Anteayer los abuelos regalaron/*regalarán un coche muy caro al nieto. |
| 89a | Anteayer el mecánico reparó/*repararon/*reparaste el coche de época de Javier. |
| 89b | Los mecánicos anteayer repararon/*repararán el coche de época de Javier. |
| 89c | El mecánico anteayer reparó/*repararon/*reparaste el coche de época de Javier. |
| 89d | Anteayer los mecánicos repararon/*repararán el coche de época de Javier. |
| 90a | Ayer temprano el accionista retiró/*retiraron/*retiraste la propuesta de acuerdo. |
| 90b | Los accionistas ayer temprano retiraron/*retirarán la propuesta de acuerdo. |
| 90c | El accionista ayer temprano retiró/*retiraron/*retiraste la propuesta de acuerdo. |
| 90d | Ayer temprano los accionistas retiraron/*retirarán la propuesta de acuerdo. |
| 91a | El mes pasado el supervisor reunió/*reunieron/*reuniste a todos los arquitectos del proyecto. |
| 91b | Los supervisores el mes pasado reunieron/*reunirán a todos los arquitectos del proyecto. |
| 91c | El supervisor el mes pasado reunió/*reunieron/*reuniste a todos los arquitectos del proyecto. |
| 91d | El mes pasado los supervisores reunieron/*reunirán a todos los arquitectos del proyecto. |
| 92a | Ayer al mediodía el panadero del barrio sacó/*sacaron/*sacaste sólo pan integral del horno. |
| 92b | Los panaderos del barrio ayer al mediodía sacaron/*sacarán sólo pan integral del horno. |
| 92c | El panadero del barrio ayer al mediodía sacó/*sacaron/*sacaste sólo pan integral del horno. |
| 92d | Ayer al mediodía los panaderos del barrio sacaron/*sacarán sólo pan integral del horno. |
| 93a | Anoche el socorrista salvó/*salvaron/*salvaste a un torpe surfista andaluz. |
| 93b | Los socorristas anoche salvaron/*salvarán a un torpe surfista andaluz. |
| 93c | El socorrista anoche salvó/*salvaron/*salvaste a un torpe surfista andaluz. |
| 93d | Anoche los socorristas salvaron/*salvarán a un torpe surfista andaluz. |
| 94a | Hace una semana el estudiante supo/*supieron/*supiste los resultados del examen. |
| 94b | Los estudiantes hace una semana supieron/*sabrán los resultados del examen. |
| 94c | El estudiante hace una semana supo/*supieron/*supiste los resultados del examen. |
| 94d | Hace una semana los estudiantes supieron/*sabrán los resultados del examen. |
| 95a | El otoño pasado el suplente sustituyó/*sustituyeron/*sustituiste a las profesoras en baja por maternidad. |
| 95b | Los suplentes el otoño pasado sustituyeron/*sustituirán a las profesoras en baja por maternidad. |
| 95c | El suplente el otoño pasado sustituyó/*sustituyeron/*sustituiste a las profesoras en baja por maternidad. |
| 95d | El otoño pasado los suplentes sustituyeron/*sustituirán a las profesoras en baja por maternidad. |
| 96a | Ayer a medianoche el peregrino agotado terminó/*terminaron/*terminaste el camino de Santiago. |
| 96b | Los peregrinos agotados ayer a la medianoche terminaron/*terminarán el camino de Santiago. |
| 96c | El peregrino agotado ayer a la medianoche terminó/*terminaron/*terminaste el camino de Santiago. |
| 96d | Ayer a medianoche los peregrinos agotados terminaron/*terminarán el camino de Santiago. |
| 97a | Hace un mes la aprendiza trabajó/*trabajaron/*trabajaste con un famoso peluquero colombiano. |
| 97b | Las aprendizas hace un mes trabajaron/*trabajarán con un famoso peluquero colombiano. |
| 97c | La aprendiza hace un mes trabajó/*trabajaron/*trabajaste con un famoso peluquero colombiano. |
| 97d | Hace un mes las aprendizas trabajaron/*trabajarán con un famoso peluquero colombiano. |
| 98a | El otoño pasado el abogado civil trasladó/*trasladaron/*trasladaste la oficina a otro edificio. |
| 98b | Los abogados civiles el otoño pasado trasladaron/*trasladarán la oficina a otro edificio. |
| 98c | El abogado civil el otoño pasado trasladó/*trasladaron/*trasladaste la oficina a otro edificio. |
| 98d | El otoño pasado los abogados civiles trasladaron/*trasladarán la oficina a otro edificio. |
| 99a | El año pasado el ginecólogo utilizó/*utilizaron/*utilizaste una nueva técnica guiada por ecografía. |
| 99b | Los ginecólogos el año pasado utilizaron/*utilizarán una nueva técnica guiada por ecografía. |
| 99c | El ginecólogo el año pasado utilizó/*utilizaron/*utilizaste una nueva técnica guiada por ecografía. |
| 99d | El año pasado los ginecólogos utilizaron/*utilizarán una nueva técnica guiada por ecografía. |
| 100a | El año pasado el fotógrafo viajó/*viajaron/*viajaste por África para retratar la pobreza. |
| 100b | Los fotógrafos el año pasado viajaron/*viajarán por África para retratar la pobreza. |
| 100c | El fotógrafo el año pasado viajó/*viajaron/*viajaste por África para retratar la pobreza. |
| 100d | El año pasado los fotógrafos viajaron/*viajarán por África para retratar la pobreza. |
